# Discovering Structure-Adaptive Oxides for Embedded Epitaxial Growth of Perovskite Nanocrystals

**DOI:** 10.1021/jacs.5c22209

**Published:** 2026-01-27

**Authors:** Yaxin Cao, Xicheng Wang, Weilin Zheng, Fengjun Chun, Zhifeng Xing, Yang Guo, Xiaohe Wei, Jiangkun Chen, Shuohan Li, Yongzheng Fang, Feng Wang

**Affiliations:** † Department of Materials Science and Engineering, 53025 City University of Hong Kong, 83 Tat Chee Avenue, Kowloon 999077, Hong Kong SAR, China; ‡ School of Materials and Energy, 12426 Lanzhou University, Lanzhou 730000, Gansu, China; § School of Materials and Packaging Engineering, Fujian Polytechnic Normal University, Fuzhou 350300, Fujian, China; ∥ School of Materials Science and Engineering, Shanghai Institute of Technology, Shanghai 201418, China; ⊥ Hong Kong Institute for Clean Energy, City University of Hong Kong, 83 Tat Chee Avenue, Kowloon 999077, Hong Kong SAR, China

## Abstract

Developing heterostructures presents a promising approach to enhance the performance of lead halide perovskite CsPbX_3_ (X = Cl, Br, I) nanocrystals (NCs) for optoelectronic applications. Given their rich variety and extreme stability, oxide crystals are appealing candidates for integration with CsPbX_3_ to expand their applications. However, heterostructural modification of CsPbX_3_ with oxides remains a daunting challenge due to the substantial lattice mismatch. This study presents a strategy for constructing CsPbBr_3_-in-oxide heterostructures under substantially mismatched lattice parameters by leveraging the structure-adaptive feature of rationally selected host materials. Our investigations reveal that complex oxide crystals comprising appropriate combinations of large and small cations can accommodate considerable misfit strain, thereby facilitating the epitaxial growth of dispersed CsPbBr_3_ NCs within the crystal lattice. Notably, the oxide matrix can effectively protect the CsPbBr_3_ NCs against water and heat, simultaneously enabling extended optical tuning through lanthanide doping. These findings provide valuable insights into heterostructural engineering of functional materials, thus representing a novel paradigm for the development and application of perovskite nanocrystal-based materials.

## Graphical abstract

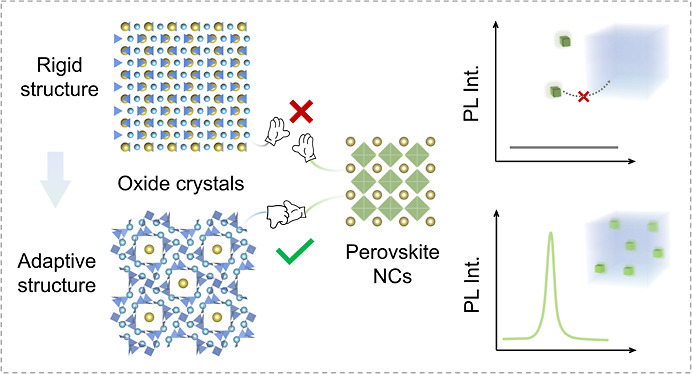

## Introduction

In the realm of material design, heterostructures have emerged as a Frontier area of research. These structures, which integrate two crystalline materials with coherent lattices, offer enhanced electronic and optical properties.^1–4^ For instance, the epitaxial growth between classic II–VI semiconductors allows for the modulation of band structures, thereby tuning carrier transport properties to cater for specific applications.^5–10^ The epitaxial growth of metallic heterostructures could provide well-defined spatial configurations, interfaces, and crystal phases, achieving special properties, such as high plasmon-induced hot-electron transfer quantum yield and highly efficient hydrogen evolution.^11–13^ Recently, heterostructural engineering has been increasingly used to refine the photophysical properties of perovskite NCs, which are promising in applications encompassing interactive display, radiation detection, and photovoltaics.^14–19^

In general, a reasonable lattice matching between perovskite and the target phase is necessary for the construction of epitaxial heterostructures.^11,20–23^ As a major class of functional materials, oxides present significant challenges in forming epitaxial heterostructures with perovskite due to substantial lattice mismatch. Despite enormous efforts, perovskite NCs have only been integrated into a limited number of oxide matrices, most of which are in the amorphous state.^24–28^ Due to a lack of structural and compositional tunability with crystallographic precision, these heterostructures typically provide insufficient improvements to the perovskite NCs. Therefore, a comprehensive investigation of oxide-perovskite systems from a materials design perspective is imperative.^15,29–37^

Herein, we present a general strategy for constructing oxide-perovskite heterostructures utilizing the structure-adaptive characteristics of rationally selected host materials. As shown in Scheme 1, oxide crystals dominantly composed of large cations (those with large ion radii and coordination numbers) typically feature high structural rigidity, necessitating stringent lattice matching for epitaxial growth of CsPbX_3_. In contrast, introducing a high fraction of small cations (those with smaller ion radii and coordination numbers) can yield flexible frameworks to mitigate the lattice mismatch constraints, thereby expanding the material combinations for heterostructural formation. By examining an extensive collection of oxide crystals, including borates, phosphates, and borophosphates, we establish practical guidelines for discovering structure-adaptive oxides that permit embedded epitaxial growth of perovskite NCs. In a case study, we synthesized a new class of Li_3_Cs_2_Ba_2_B_3_P_6_O_24_-CsPbBr_3_ (LCBBP-CPB) crystals with dual emission capability due to embedded perovskite NCs and substitutional lanthanide ions in the oxide lattice, of which systematic structural and spectroscopy characterizations were performed.

**Scheme 1:**
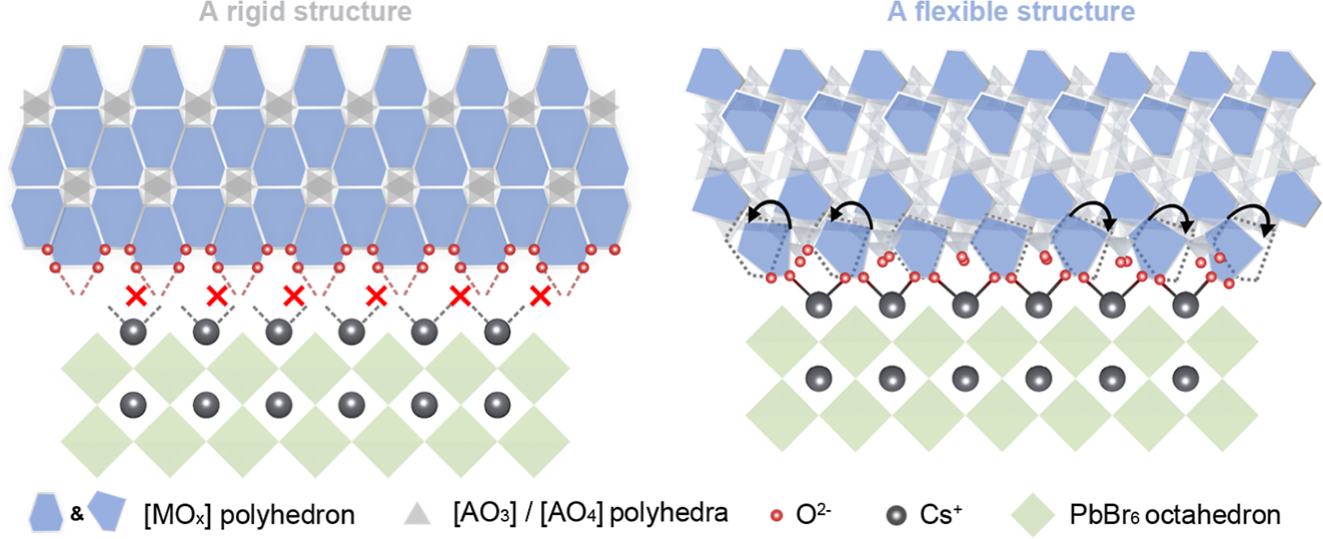
Schematic Illustration of the Interface between CsPbBr_3_ and Oxide with Rigid and Flexible Structures for Possible Epitaxial Growth^a^

a Rigid structure (left) is formed by large cations (blue [MO_*x*_] polyhedra, e.g., alkali/alkaline earth, rare earth ions) with high coordination numbers (CN ≥ 6) through edge-/face-sharing polyhedral connectivity, enforcing strict lattice-matching rules for CsPbX_3_ nanocrystal (NC) epitaxy. In contrast, flexible structure (right) comprising grey [AO_3_]/[AO_4_] polyhedra building blocks of small cations (e.g., B^3+^, P^5+^) with low coordination numbers (CN = 3/4) via corner-sharing configurations, enabling dynamic lattice adjustments to accommodate localized distortions induced by CsPbX_3_ NCs while preserving structural integrity.

## Results and Discussion

Our studies focus on borates, phosphates, and borophosphates owing to their intrinsic flexibility associated with [BO_3_]/[BO_4_] and [PO_4_] units. These units could form corner- or edge-sharing frameworks (Figure 1a), contributing to the adaptable crystal structure in the corresponding oxides.^38–40^ To enable epitaxy, large-radius cations, such as Rb^+^/Na^+^ (alkali metals) and Ba^2+^/Sr^2+^ (alkaline earth metals), are also included in the oxide matrix for interfacing with perovskite NCs (Figure 1a).^20,41^ Notably, these elements can form a large family of complex oxides with diverse structural features. To establish a practical guideline for the rational selection of host matrices suitable for epitaxial growth of perovskite NCs, we synthesized an extensive collection of complex oxide crystals in the presence of CsPbBr_3_ NCs. The materials were classified according to the structural units ([BO_3_]/[BO_4_] or [PO_4_]) and the types of large cations (monovalent, M^+^, or polyvalent, M^*n*+^), as presented in Figure S1. The detailed information for all the prepared samples is collected in Supporting Information (Table S1 and Figures S2–S20), with the synthesis protocol (take LCBBP-CPB for instance) shown in Figure S21. The synthesis utilized an in situ growth strategy, which provides more effective encapsulation than approaches incorporating presynthesized CsPbBr_3_ nanocrystals,^42^ as the elimination of organic ligands enhances interfacial compatibility and structural stability (Figure S22). As summarized in Table S2, the successful formation of epitaxial heterostructures is not positively correlated with the degree of lattice matching between CsPbBr_3_ and the oxide matrix.

**Figure 1:**
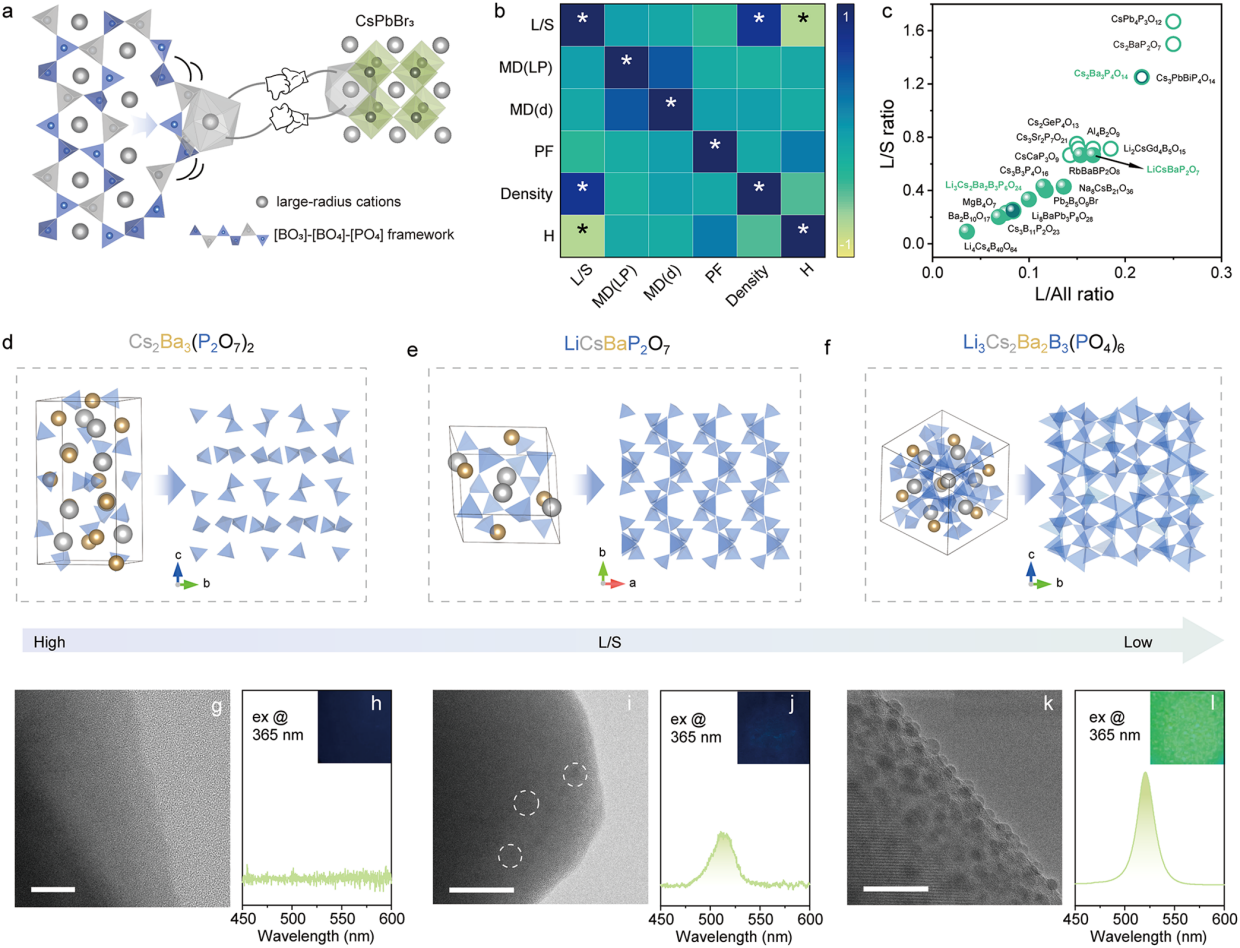
Construction and characterization of host-CsPbBr_3_ heterostructure materials. (a) A scheme illustrating the structural adaptability in a flexible structure for the epitaxial growth of CsPbBr_3_ NCs. (b) Pearson correlation matrix of structural parameters: L/S ratio, lattice matching degree (MD­(LP)), interplanar spacing matching degree (MD­(d)), packing factor (PF), crystal density, and heterostructure formation (H). Asterisks indicate that the correlation coefficients are statistically significant. (c) Correlation of PL behavior of the as-synthesized samples with large cation proportion (calculation details are shown in Table S4). (d–f) Crystal structures of (d) Cs_2_Ba_3_(P_2_O_7_)_2_, (e) LiCsBaP_2_O_7_, and (f) Li_3_Cs_2_Ba_2_B_3_(PO_4_)_6_ with varying L/S ratios. The right parts of each figure show the flexible building blocks extracted from the corresponding related crystal structures. (g–l) TEM images and corresponding PL spectra for (g,h) Cs_2_Ba_3_(P_2_O_7_)_2_, (i,j) LiCsBaP_2_O_7_, and (k,l) Li_3_Cs_2_Ba_2_B_3_(PO_4_)_6_. Scale bars: 20 nm. The insets are the corresponding photographs of the samples under 365 nm excitation.

This was the impetus for our deeper inquiry into the formation mechanisms of epitaxial heterostructures. Accordingly, a Pearson correlation analysis was conducted to elucidate the relationship between fundamental structural parameters of materials and heterostructure formation (H), as deduced from perovskite luminescence. The correlation matrix (Figure 1b) reveals a strong negative correlation (*r* = −0.76) between the mole ratio of large to small cations (L/S) and H, underscoring its critical role in governing the epitaxial process. Details regarding the definitions and descriptions of the Pearson correlation analysis are provided in Table S3. The detailed definitions and calculations of the relevant parameters are provided in eqs S1–S7 and Tables S4–S8. Figure 1c correlates the photoluminescence (PL) behavior of the as-synthesized samples with L/S, in which solid and open circles denote emissive host-CsPbBr_3_ heterostructures and nonemissive samples, respectively.

To shed light on the effect of L/S ratio, we analyzed a series of chemically similar compounds consisting of Cs_2_Ba_3_(P_2_O_7_)_2_, LiCsBaP_2_O_7_, and Li_3_Cs_2_Ba_2_B_3_(PO_4_)_6_ with decreasing L/S ratios of 5/4, 2/3, and 1/3 by incorporating Li^+^ and B^3+^. The related samples were marked in green font in Figure 1c. As illustrated in Figure 1d, a high proportion of large Cs^+^ and Ba^2+^ ions in Cs_2_Ba_3_(P_2_O_7_)_2_ construct a close-packed large-cation polyhedral framework, resulting in a rigid structure due to spatially isolated small-cation polyhedra (i.e., [PO_4_] tetrahedra in the form of corner-shared [P_2_O_7_] units). Progressive increase of the small-cation contents in LiCsBaP_2_O_7_ and Li_3_Cs_2_Ba_2_B_3_(PO_4_)_6_ (Figure 1e,f) results in the interconnection of small-cation units ([PO_4_], [BO_4_], and [LiO_4_]). Along with the structural evolution, perovskite nanoparticles gradually formed within the oxide matrix as evidenced by TEM images (Figure 1g,i,k), resulting in bright green emissions (Figure 1h,j,l). These results indicate that a small L/S ratio (<0.67) favors the framework formation among flexible building blocks, contributing to structural adaptability that enables the embedded epitaxial growth of perovskite NCs. Particularly, Li_3_Cs_2_Ba_2_B_3_(PO_4_)_6_ exhibits the highest structural flexibility owing to the largest density of small cations that forms a three-dimensional corner-sharing mode (Figure 1f).

To validate the formation of an epitaxial heterostructure, the Li_3_Cs_2_Ba_2_B_3_(PO_4_)_6_-CsPbBr_3_ heterostructure (denoted as LCBBP-CPB) was characterized in detail. Scanning electron microscope (SEM) combined with energy dispersive X-ray spectroscopy (EDS) elemental mapping (Figure 2a) demonstrates uniform dispersion of all elements, particularly Cs, Pb, and Br, throughout the oxide particle. Complementary X-ray photoelectron spectroscopy (XPS) analysis (Figure S23) confirms the presence of Li. Local TEM imaging in Figure 2b reveals ∼5 nm nanoparticles dispersed in a well-crystallized matrix, which is demonstrated by the corresponding fast Fourier transform (FFT) pattern in Figure 2c. Further inverse fast Fourier transform (IFFT) analysis of the specified region (Figure 2d) resolves an interplanar spacing of 0.42 nm, consistent with the (221) plane of LCBBP. The high-resolution TEM (HRTEM) image (Figure 2e) exhibits moiré fringes related to the shape of nanoparticles, and the enlarged image with higher contrast is presented in Figure 2f. The corresponding FFT pattern (Figure 2g) contains two discrete reflection sets assigned to LCBBP (421) and CsPbBr_3_ (211) planes, demonstrating the formation of LCBBP-CPB heterostructures.^43^ Figure 2h reveals a coherent LCBBP/CsPbBr_3_ interface. The FFT pattern in Figure 2i (for the boxed region in Figure 2h) and the corresponding IFFT analysis of this region (Figure 2j) identify a transitional zone (interfacial region). In detail, the LCBBP interplanar spacing adapts to accommodate the CsPbBr_3_ lattice, signifying the epitaxial growth nature. A schematic in Figure 2k illustrates the local structural adaptation of LCBBP, contributing to the epitaxial interfacing with CsPbBr_3_.

**Figure 2:**
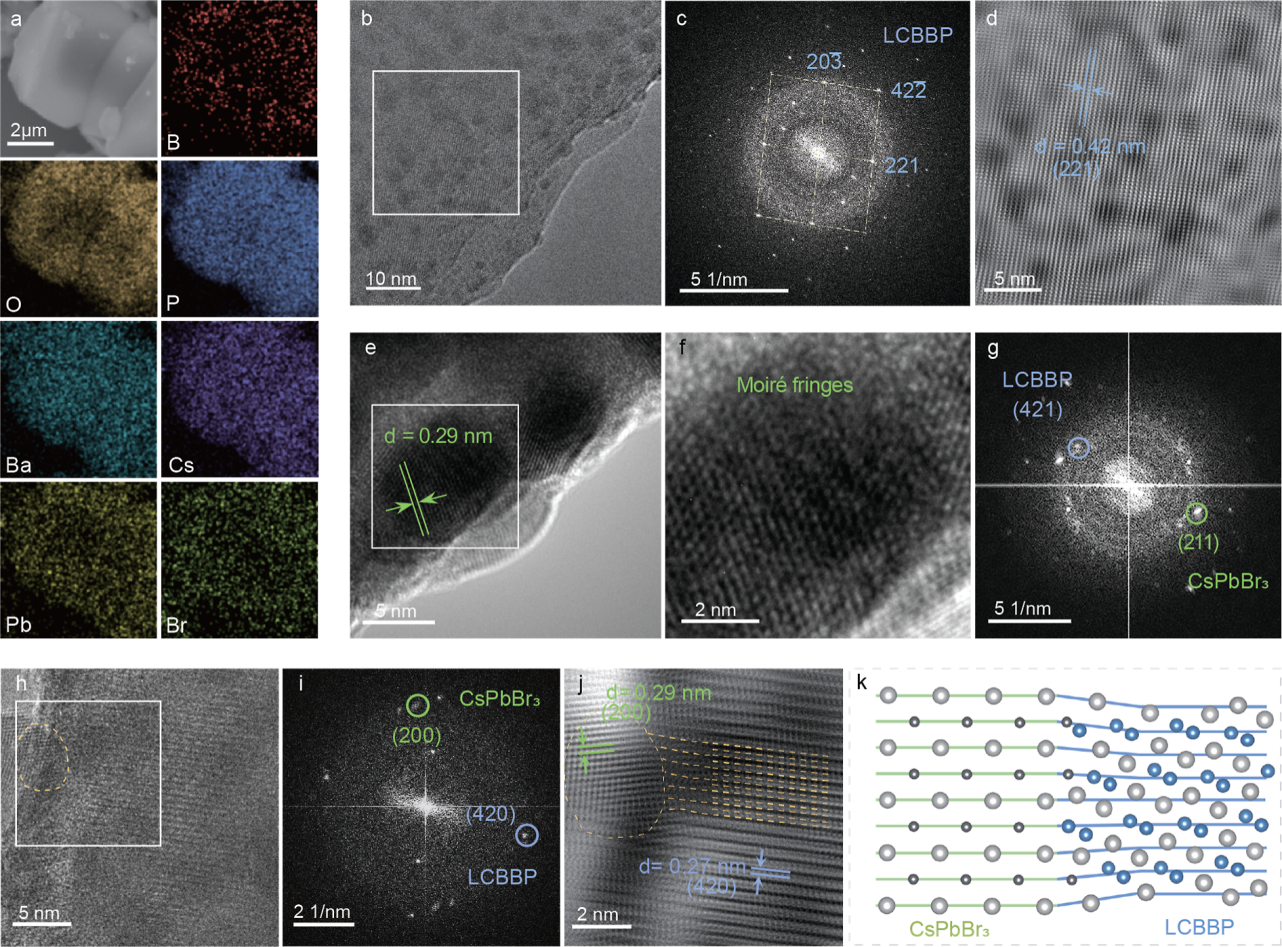
Comprehensive characterization of the LCBBP-CPB heterostructure. (a) EDS elemental mapping of the LCBBP-CPB particles. (b) TEM image with the FFT pattern (c,d) IFFT image of the selected area in (b). (e) HRTEM image with magnified moiré fringes (f) and the corresponding FFT pattern (g) of the selected area in (e). (h) HRTEM image of the heterostructure with the FFT pattern (i) and IFFT image (j) of the selected area in (h). (k) Schematic of lattice matching between LCBBP and CsPbBr_3_. The large cations are shown in different colors: Cs^+^ (light gray), Pb^2+^ (dark gray), and Ba^2+^ (blue).

Building on the epitaxial heterostructure analysis, we probed the crystallographic characteristics of the materials through accurate structural characterization strategies. X-ray diffraction (XRD) measurement confirmed the pure and well-crystallized phase of LCBBP–CsPbBr_3_ (Figure 3a). The absence of CsPbBr_3_-related peaks can be ascribed to the relatively weak diffraction of NCs with a small size and low content. The related Rietveld refinement^44^ of the LCBBP matrix yielded a weighted profile *R*_wp_ of 2.96% (Tables S9 and S10). Four types of large cation sites in LCBBP are confirmed, i.e., Ba1 (CN = 6), Ba2 (CN = 9), Cs1 (CN = 9), and Cs2 (CN = 9), respectively. Raman spectroscopy (Figure 3b) detected characteristic vibrational modes at 1063 and 629 cm^–1^, verifying [PO_4_] tetrahedral units^45^ and P–O–B polyanionic rings^46^ within the LCBBP lattice. Structural analysis reveals a cation-ordered channel framework wherein interconnected small-cation polyhedra define periodic channels housing large-cation polyhedra (Figure 3c). This architectural motif recurs systematically across other synthesized host matrices. Arising from its polyhedral units, the structural flexibility is proposed to facilitate heterostructure formation by accommodating lattice mismatch between the oxide matrix and CsPbBr_3_ NCs.

**Figure 3:**
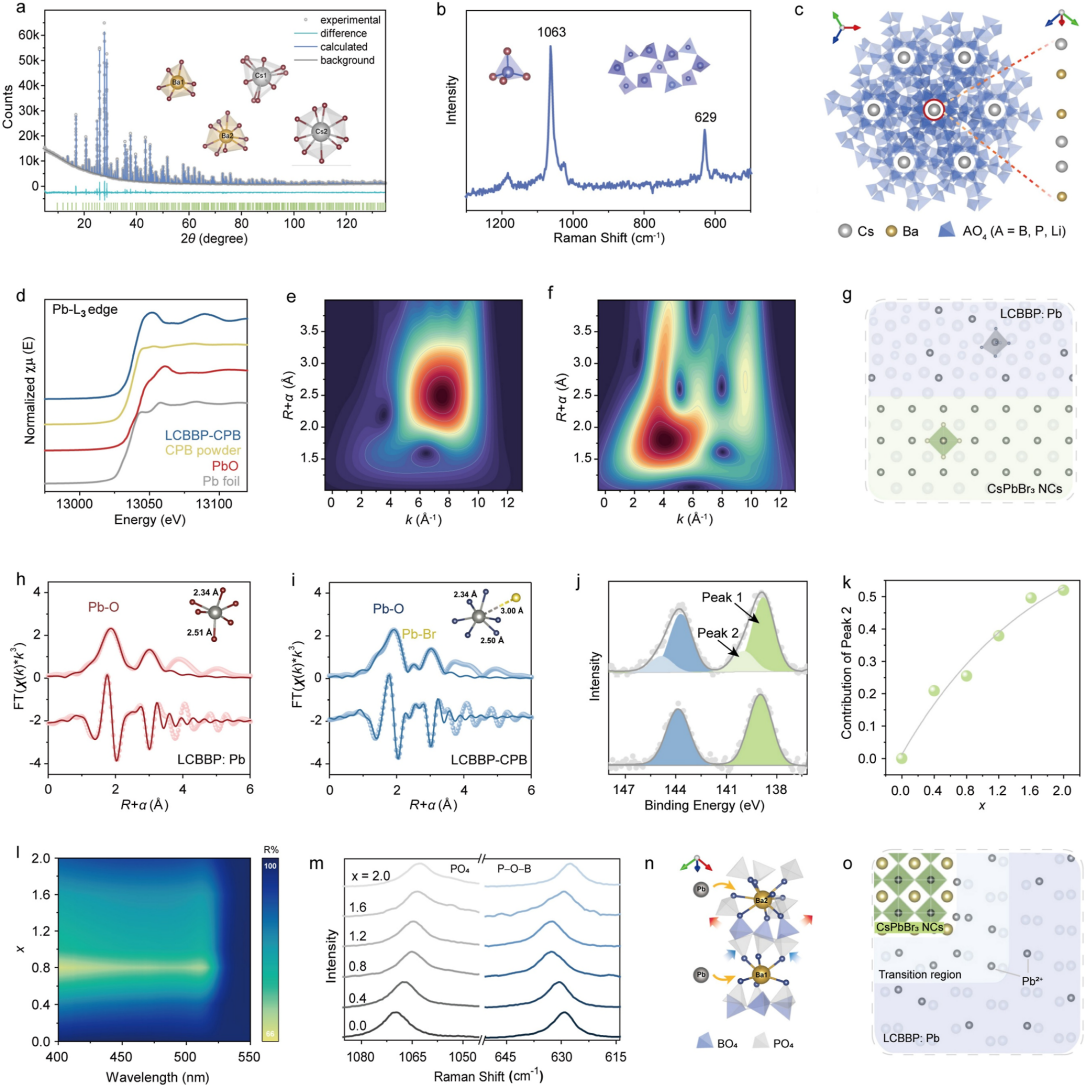
Local environment revolution of the LCBBP-CPB heterostructure construction. (a) XRD refinement (inset: large cation-coordination in LCBBP), (b) Raman spectrum (inset: [PO_4_] and the [P–O–B] rings in LCBBP), (c) crystal structure of the LCBBP matrix. (d) Pb L_3_-edge XANES spectra of LCBBP-CPB, compared with CsPbBr_3_ powder, Pb foil, and PbO as references. Wavelet transform analysis of the *k* ^3^-weighted EXAFS data for (e) CsPbBr_3_ and (f) LCBBP-CPB. (g) Schematic diagram showing the existence forms of Pb in the LCBBP matrix and CsPbBr_3_ NCs. FT magnitude and real component of the *k* ^3^-weighted Pb L_3_-edge EXAFS spectra in R-space, along with fitting curves, for (h) LCBBP and (i) LCBBP-CPB; the inset illustrates the coordination status of Pb in the two samples. (j) Pb 4f XPS spectra of LCBBP (lower) and LCBBP-CPB (upper). (k) Integrated intensity ratios of the higher-energy sub-band in Pb high-resolution XPS spectra as a function of Pb content (*x*). (l) DRS spectra as a function of Pb content (*x*). (m) Raman spectra of the series samples. (n) Schematic diagram of Pb-coordinated structures associated with [PO_4_] and P–O–B rings. (o) Schematic of Pb chemical environments in LCBBP-CPB.

To elucidate the local environment of Pb in LCBBP-CPB for a better understanding of the heterostructure construction, X-ray absorption near edge structure (XANES) and extended X-ray absorption fine structure (EXAFS) analyses were conducted. The Pb L_3_-edge XANES spectrum of LCBBP-CPB resembles that of PbO, indicating that Pb exists in a divalent oxidation state with Pb–O coordination dominance (Figure 3d). EXAFS wavelet analysis of CsPbBr_3_ (Figure 3e) reveals a prominent feature at (*R* + α) = 2.5 Å, corresponding to Pb–Br bonding. For LCBBP-CPB (Figure 3f), intense peaks at *R* ∼1.9 Å and ∼3.0 Å are attributed to the first- and second-neighbor Pb–O distances, respectively. Enhanced intensity in the *R* range of 1.5–3.5 Å (*k* range: 6–10 Å^–1^) suggests the coexistence of Pb–Br and Pb–O bonding nature in LCBBP-CPB (Figure S24 and S25). The results suggest that excess Pb in PbBr_2_ as the raw material for CsPbBr_3_ NCs was incorporated into the LCBBP lattice (Figure 3g). EXAFS fitting of Pb-substituted LCBBP (LCBBP:Pb) and LCBBP-CPB (Figures 3h,i and S26) reveals similar Pb–O coordination environments, with a first shell coordination number of six at ∼1.90 Å and a second shell of three at ∼3.0 Å (Tables S11 and S12). The path of Pb–Br was introduced in the fitting for LCBBP-CPB, and it suggested that Pb–Br might contribute to the second shell with a pretty small proportion, mainly attributed to the Pb–Br bonds near the interface in the heterostructure. XRD refinement indicates two Ba sites in LCBBP, with Pb preferentially substituting Ba1 (nearest oxygen number of 6) before occupying Ba2 (nearest oxygen number of 9) (Table S13). Thus, the presence of Ba^2+^ ions (large cations, with a suitable coordination environment) in the host is essential for the framework’s flexibility, that is, accommodating Pb-substitution and facilitating CsPbBr_3_ NCs growth.

To further probe the structural characteristics of the LCBBP-CPB heterostructure, we conducted a comprehensive investigation combining multiple spectroscopic techniques. A comparative analysis of XPS spectra (Figure 3j) reveals additional higher-binding-energy peaks of Pb at 140.0 and 144.9 eV in LCBBP-CPB, distinct from LCBBP:Pb (138.8 and 143.8 eV). The additional peaks are ascribed to Pb–O bonding at the interfacial region between CsPbBr_3_ NCs and LCBBP, which yields higher energy due to misfit strain. Systematic XPS analysis of samples with varying PbBr_2_ molar ratios (*x*) demonstrated a progressive increase in the higher-binding-energy peak (marked as Peak 2 and the one with lower energy is marked as Peak 1) intensity with *x* (Figures 3k and S27), confirming its correlation with Pb incorporation. Based on the above analysis, it can be inferred that the proportion of the higher-binding-energy peak should be related to the content of CsPbBr_3_ NCs.

The presence of CsPbBr_3_ NCs in the LCBBP matrix was further identified by the absorption characteristics of CsPbBr_3_ NCs using diffuse reflectance spectroscopy (DRS).^47^ The additional 300–520 nm absorption feature in the LCBBP-CPB heterostructure compared to the LCBBP host (with its absorption band centered at ∼260 nm, which is consistent with the calculated bandgap, as shown in Figures S28 and S29) is assigned to the intrinsic absorption of CsPbBr_3_. The series DRS spectra in Figure 3l reveal that the CsPbBr_3_ absorption reached saturation at *x* = 0.8, suggesting a limitation in NC growth beyond this point. This observation raises an apparent contradiction with the monotonic increase in XPS peak intensity. To resolve this, Raman spectroscopy was employed (Figure 3m). The continuous peak shift in the antisymmetric [PO_4_] stretching mode (∼1060 cm^–1^) and symmetric P–O–B vibrations (∼629 cm^–1^) indicates that Pb incorporation could modulate the LCBBP framework. Specifically, the ∼629 cm^–1^ peak shifts to higher frequencies first and subsequently back to lower frequencies as Pb content increases. This could be related to the local lattice strain change on P–O–B units generated by Pb’s substitution for Ba1 (compressive stress) and Ba2 (tensile stress) in sequence. The simplified structural diagram is shown in Figure 3n. Another fact is that the sample begins to be Pb-rich (less structure-adaptable space, halting CsPbBr_3_ NC growth) instead of Ba-rich (with more structure-adaptable space, facilitating heterostructure formation) after *x* > 0.8 (approaching complete substitution of Ba1 by Pb), which changes the stress type in further Pb–Ba-substitution. The monotone increasing proportion of the higher-binding-energy peak after *x* > 0.8 in Figure 3k should mainly be induced by the expanded interfacial region of the heterostructure. Collectively, the adaptable local structure of LCBBP enables epitaxial growth of CsPbBr_3_ NCs, while Pb-for-Ba substitution triggers collective structural modification, consequently reducing lattice strain at the interface. Specifically, a unique feature lies in the full substitutability of Ba sites by Pb (Figure S30), making it a good candidate for hosting CsPbBr_3_ NCs, which can also be an inspiration for the selection of an appropriate matrix. Based on these findings, we propose a structural model for LCBBP-CPB (Figure 3o). A transition region (corresponding to the structural adaptive range) is located outside the CsPbBr_3_ NC surface, which contains Pb^2+^ ions with higher energy (compared to the substitutional Pb^2+^ ions in LCBBP) induced by lattice strain, connecting the nanoparticles with the matrix. Our results provide strong evidence for the feasibility of structural adaptation of flexible frameworks in constructing such heterostructures. Moreover, the crystallographic order facilitates comprehensive characterization and mechanistic elucidation (e.g., atomic-scale structural analyses) of the oxide-perovskite epitaxial heterostructure.

The interfacial engineering in LCBBP-CPB epitaxial heterostructure modulates the properties of CsPbBr_3_. Notably, the heterostructure exhibits a remarkable lifetime extension (62 ns) compared to colloidal CsPbBr_3_ NCs (5 ns, Figure 4a), indicating that the epitaxial heterostructure may lead to NCs with improved structural integrity.^48–52^ Further correlation of the Pb–O signature with optoelectronic properties in LCBBP-CPB reveals a nonmonotonic dependence of the photoluminescence quantum yield (PLQY) and carrier lifetime on the Pb–O incorporation level (Figure S31). An optimal window is observed at a precursor ratio of *x* ≈ 0.4, where a balanced interface favors defect suppression and coherent strain accommodation, thereby maximizing the PLQY. Beyond this point, excessive Pb–O incorporation could introduce detrimental defects and strain, which degrade the luminescence efficiency despite a prolonged apparent carrier lifetime due to enhanced trapping.^2,8,9^ This optimal Pb–O coordination window could provide guidance for precursor stoichiometry in achieving high-performance heterostructures.

**Figure 4:**
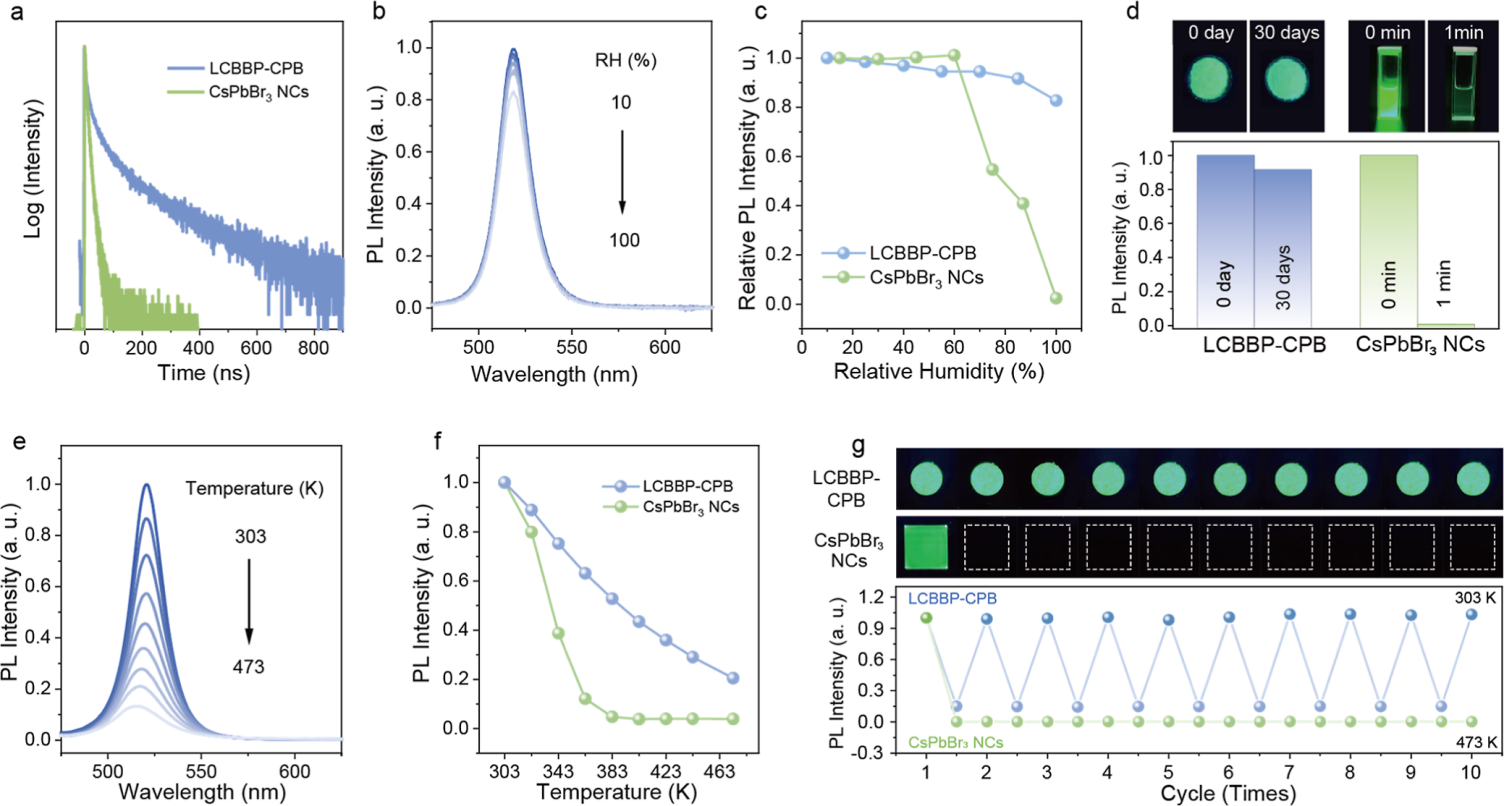
(a) Decay curves of LCBBP and LCBBP-CPB. (b) Humidity-dependent PL spectra of LCBBP-CPB and the (c) comparison with CsPbBr_3_ NCs. (d) Water stability comparison: photographs (top) and retained PL intensity (bottom) of LCBBP-CPB and CsPbBr_3_ NCs after immersing in water (30 days vs 1 min). (e) Temperature-dependent PL spectra of LCBBP-CPB and the (f) comparison with CsPbBr_3_ NCs. (g) Thermal cycling stability (303–473 K) of LCBBP-CPB, with insets showing temperature-dependent color changes for LCBBP-CPB powder and CsPbBr_3_ NCs on glass substrates. Excitation wavelength for all the test results in (b–g) was fixed at 365 nm.

The oxide matrix also provides effective protection for the perovskite NCs against environmental factors. As shown in Figure 4b, the luminescence of LCBBP-CPB shows a marginal decline with increasing relative humidity (RH), and the PL intensity recovers nearly completely upon drying (Figure S32). Figure 4c compares the humidity response of LCBBP-CPB with CsPbBr_3_ NCs, where the latter’s intensity retention below 60% RH may relate to water-induced defect passivation.^53,54^ Consistent with methods commonly reported in the literature, LCBBP-CPB maintained 92% of its initial intensity after thoroughly mixing with deionized (DI) water by shaking for 1 min, followed by 30 days of continuous immersion in DI water. In stark contrast, CsPbBr_3_ NCs dispersed in cyclohexane underwent complete PL quenching after 1 min of mixing with DI water. Relevant results and photographs are presented in Figure 4d. Furthermore, the LCBBP-CPB heterosystem also exhibits excellent thermal stability. Figure 4e presents the temperature-dependent PL spectra of LCBBP-CPB from 303 to 473 K. Unlike CsPbBr_3_ NCs in many other matrices (e.g., some glass and molecular sieves)^55–57^ exhibiting a rapid decrease in PL intensity with increasing temperature, the PL thermal quenching of LCBBP-CPB is appreciably mitigated compared to CsPbBr_3_ NCs (Figures 4f and S33). Further tests demonstrate that the emission intensity of LCBBP-CPB under 365 nm excitation recovered upon cooling back to room temperature, exhibiting negligible performance degradation over 10 heating–cooling cycles (Figure 4g). This contrasts sharply with CsPbBr_3_ NCs, which undergo complete degradation after the initial temperature ramp to 473 K, as visually confirmed by the comparative photographs (Figure 4g, top panel).

In a further set of experiments, we demonstrated that the two components in the heterostructure can be independently engineered for flexible optical tuning. By virtue of its structure-adaptive feature, the LCBBP matrix can host a series of mixed-halide perovskite NCs [i.e., CsPb­(Br,I)_3_; Br/I ratio = 3/0, 2.1/0.9, 1.5/1.5, 0.75/2.25, and 0/3], rendering tunable emission ranging from 520 to 700 nm (Figure 5a). Furthermore, the LCBBP host also permits doping of luminescent lanthanide ions (e.g., Eu^3+^) for dual-emission tuning by precisely controlling the doping concentration (Figure 5b). Notably, the Eu^3+^-doped heterostructure exhibits distinct emission colors under different excitation wavelengths (Figure 5c). Particularly, under 350 nm excitation, the green emission from CsPbBr_3_ NCs consistently dominates the luminescence, irrespective of Eu^3+^ doping (Figure S34). In contrast, the emission progressively shifts from green to yellow (or red depending on the Eu^3+^ concentration) by excitation at shorter or longer wavelengths, at which characteristic absorptions of Eu^3+^ are present (e.g., 262 nm attributed to the charge transfer band, 395 nm assigned to ^7^F_0_–^5^L_6_ transition). Besides, the emission color of the Eu^3+^-doped LCBBP-CPB demonstrates high sensitivity to temperature (represented by LCBBP-Y01, Figures 5d and S35), stemming from the higher luminescence thermal stability of Eu^3+^ ions than perovskite NCs.

**Figure 5:**
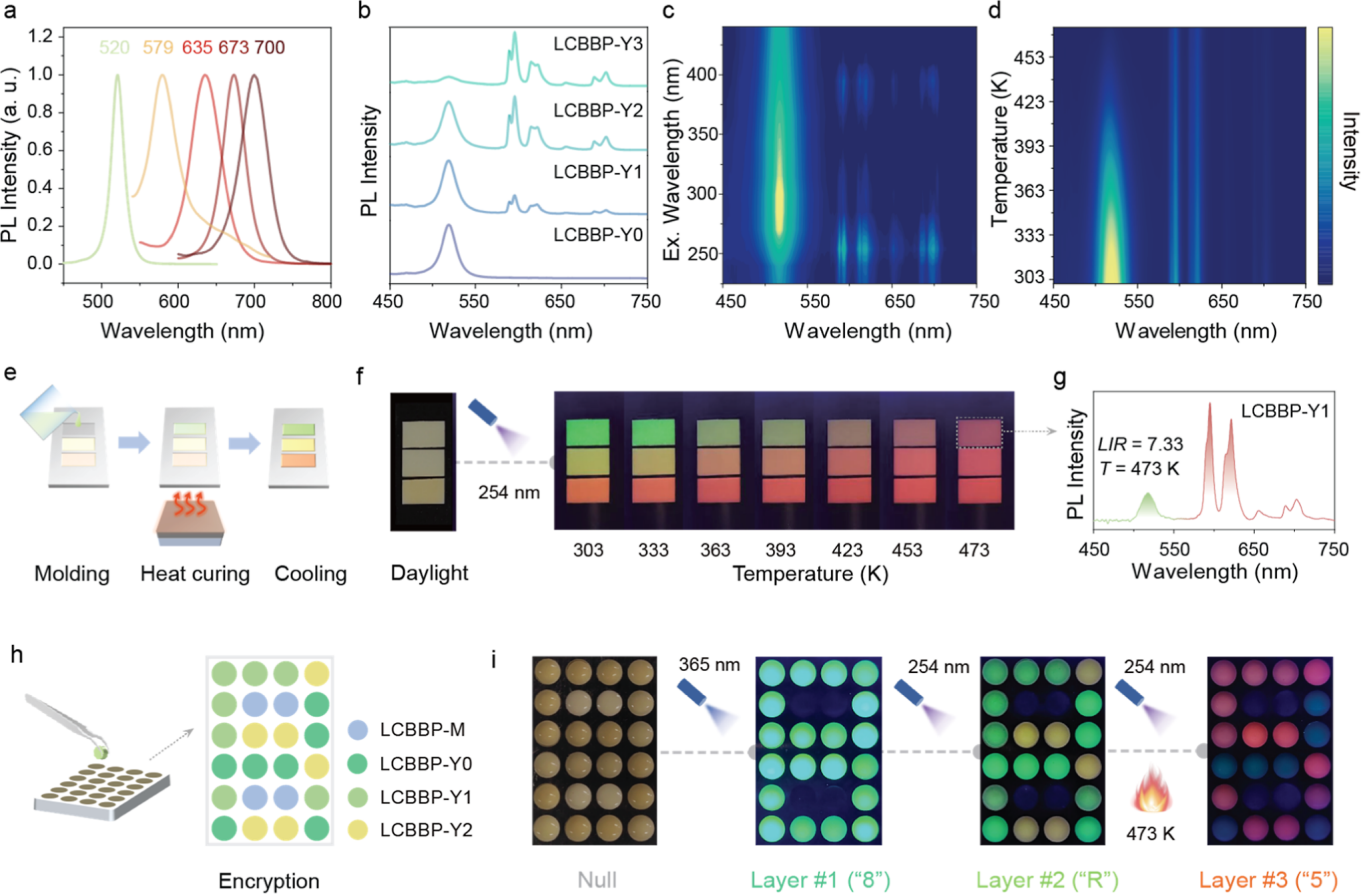
Multicolor tuning of LCBBP-CPB. (a) PL spectra for LCBBP–CsPbX_3_ (X = Br and I, with Br/I ratios of 3/0, 2.1/0.9, 1.5/1.5, 0.75/2.25, and 0/3, respectively). (b) PL spectra of LCBBP-CPB: *y*Eu^3+^ (*y* = 0, 10%, 20%, and 30%, denoted as LCBBP-Y0, LCBBP-Y1, LCBBP-Y2, and LCBBP-Y3, respectively) by excitation of 254 nm. (c) Excitation wavelength-dependent PL spectra of LCBBP-CPB: 10% Eu at room temperature. (d) Temperature-dependent PL spectra of LCBBP-CPB: 10% Eu under 254 nm excitation. (e) Schematic presentation of the colorimetric thermometer made of LCBBP-Y1, LCBBP-Y2 and LCBBP-Y3. (f) Colorimetric temperature indicating under 254 nm excitation (303–473 K). (g) PL spectrum for the sample indicated by a dashed box in (f). (h) Schematic of the pixelated pattern consisting of LCBBP-derived materials for information encryption. LCBBP-M represents the LCBBP matrix, which exhibits no visible emission under ultraviolet light excitation. (i) The decryption process based on three groups of photonic output codes under diverse excitation/temperature parameters.

The dual emission that can be modulated by excitation wavelength and temperature offers a promising strategy for information encryption and temperature-sensing applications. To demonstrate potential applications, a visualized colorimetric temperature indicator (CTI) was fabricated by casting a mixture of Eu^3+^-doped LCBBP-CPB and polydimethylsiloxane (PDMS) into an aluminum alloy mold through heat curing at 80 °C (Figure 5e). Under 254 nm excitation, the CTI displays orange, yellow, and green emissions from different segments at 303 K. As temperature gradually increases, these segments progressively turn red due to their differential colorimetric responses to temperature (Figure 5f). Notably, more accurate temperature values can be obtained by examining the emission spectrum of the sample undergoing color change, based on the luminescence intensity ratio of the green and red emissions (Figure 5g). Furthermore, a multicolor array was developed by patterning Eu^3+^-doped LCBBP-CPB in PDMS into an aluminum alloy mold (Figure 5h). The encrypted information can be deciphered layer by layer using a sequence of excitation wavelength and thermal stimulation combinations, as shown in Figure 5i.

## Conclusion

In summary, this study introduces an innovative design principle for constructing CsPbX_3_-in-oxide heterostructures under substantial lattice mismatch. Through systematic correlation analysis and case studies focusing on borates, phosphates, and borophosphates, we demonstrate that complex oxide crystals comprising appropriate combinations of large and small cations can accommodate considerable misfit strain, thereby facilitating the epitaxial growth of dispersed CsPbBr_3_ NCs within the crystal lattice. Besides, we present the first atomic-scale characterization and mechanistic analysis of local structure within LCBBP-CPB as a model system. Notably, the LCBBP matrix can accommodate a series of mixed-halide perovskite NCs, simultaneously permitting lanthanide doping for dual modulation of emission properties by precise control of excitation wavelength and temperature. Our findings thus highlight a new paradigm in the development of perovskite nanocrystal-based heterostructured materials, which not only addresses fundamental challenges in heterostructure synthesis but also opens fresh avenues for the creation of advanced functionalities.

## Supplementary Material



## Notes

Experimental section for synthesis, characterization of oxide-perovskite materials, and computational details, Pearson correlation analysis data sets, Rietveld refinement parameters for the LCBBP matrix, EXAFS fitting parameters, characterization data including XRD patterns, TEM images, and optical properties of various oxide systems, additional spectroscopic analyses (XPS, Raman, DRS), and band structure calculations (PDF)
