# Supplementary material for: Discovering Structure-Adaptive Oxides for Embedded Epitaxial Growth of Perovskite Nanocrystals
Source: J Am Chem Soc. 2026 Jan 27;148(5):5754–63. doi: 10.1021/jacs.5c22209 (PMC12903847; doi:10.1021/jacs.5c22209)
Supplement: Supplementary file 1 [file ja5c22209_si_001.pdf]

Supporting Information

Discovering structure-adaptive oxides for embedded epitaxial growth of perovskite nanocrystals

Yaxin Cao[a], Xicheng Wang[ab]*, Weilin Zheng[ac], Fengjun Chun[a], Zhifeng Xing[a], Yang Guo[a], Xiaohe Wei[a], Jiangkun Chen[a], Shuohan Li[a], Yongzheng Fang[d]*, Feng Wang[ae]*

a: Department of Materials Science and Engineering, City University of Hong Kong, 83 Tat Chee Avenue, Kowloon 999077, Hong Kong SAR, China
b: School of Materials and Energy, Lanzhou University, Lanzhou 730000, Gansu, China
c: School of Materials and Packaging Engineering, Fujian Polytechnic Normal University, Fuzhou 350300, Fujian, China
d: School of Materials Science and Engineering, Shanghai Institute of Technology, Shanghai 201418, China
e: Hong Kong Institute for Clean Energy, City University of Hong Kong, 83 Tat Chee Avenue, Kowloon 999077, Hong Kong SAR, China

Corresponding Authors
Xicheng Wang − Department of Materials Science and Engineering, City University of Hong Kong, 83 Tat Chee Avenue, Kowloon 999077, Hong Kong SAR, China
School of Materials and Energy, Lanzhou University, Lanzhou 730000, Gansu, China
ORCID: https://orcid.org/0000-0002-4310-5967 Email: wangxc@lzu.edu.cn

Yongzheng Fang − School of Materials Science and Engineering, Shanghai Institute of Technology, Shanghai 201418, China
Email: fangyongzheng@sit.edu.cn

Feng Wang − Department of Materials Science and Engineering, City University of Hong Kong, 83 Tat Chee Avenue, Kowloon 999077, Hong Kong SAR, China
Hong Kong Institute for Clean Energy, City University of Hong Kong, 83 Tat Chee Avenue, Kowloon 999077, Hong Kong SAR, China
ORCID: https://orcid.org/0000-0001-9471-4386 Email: fwang24@cityu.edu.hk

Experimental Section

Chemicals and Synthesis

Host-CPB: The host-CPB samples were synthesized via a conventional solid-state reaction method. Analytic reagents for the raw materials were precisely weighed in stoichiometric proportions as specified in **Table S1**. The mixture was then thoroughly ground using cyclohexane as a dispersing medium in a fume hood until complete evaporation of the solvent was achieved. Subsequently, the homogenized mixture was transferred to an alumina crucible and subjected to a calcination process in a muffle furnace under ambient atmosphere. The thermal treatment was performed according to the temperature profile detailed in **Table S1**, with a controlled heating rate of 5 °C/min and subsequent natural cooling to room temperature. The resulting product was finely pulverized using an agate mortar, and the resultant powder was collected for subsequent characterization and analysis.

LCBBP-CPB: The synthesis of LCBBP-CPB and the $Li_3Cs_2Ba_{2-x}Pb_xB_3P_6O_{24}$-CPB series (where *x* represents the molar ratio of $PbBr_2$ in the starting materials) was carried out following a procedure analogous to that described in the preceding section.

LCBBP-CPB: *y*Eu^{3+}: The synthesis conditions for the Eu^{3+}-doped samples were maintained identical to those employed for the preparation of LCBBP-CPB. Europium oxide (Eu_2O_3, A. R.) was utilized as the dopant source, with varying concentrations introduced to achieve different Eu^{3+} doping levels in the final products.

LCBBP-$CsPbX_3$ (X = Br, I): The growth of mixed-halide perovskite $CsPbX_3$ (X = Br, I) nanocrystals in LCBBP followed a procedure similar to that of LCBBP-CPB, which was synthesized under continuous nitrogen flow. PbI_2 (A. R.) was added to achieve Br:I molar ratios of 2.8:1.2, 2:2, 1:3, and 0:4, corresponding to different halide compositions in the final products.

Colloidal $CsPbBr_3$ NCs: The well-established hot-injection method was applied, and the detailed process was described in the reference [*Nano Lett.* 2015, 15, 6, 3692–3696].

Patterns for information encryption and colorimetric temperature indicator: Crosslinked polydimethylsiloxane (PDMS, SYLGARD 184 Kit, Dow Corning, Midland, MI, USA) was employed as the polymeric matrix for pattern fabrication. The LCBBP-CPB: *y* Eu^{3+} phosphor was uniformly dispersed in the PDMS precursor at a weight ratio of 1:1, followed by the addition of the curing agent. The mixture was thoroughly homogenized through mechanical stirring, subsequently transferred into an alumina mold, and thermally cured in an oven at 80 °C for 3 h to achieve complete crosslinking.

Physical Measurements

X-ray diffraction (XRD) patterns were collected on a Bruker D2 phaser XE-T X-ray diffractometer system by using Cu Kα radiation (λ = 1.5406 Å) with operating current and voltage of 30 kV and 10 mA, and data used for Rietveld refinements were collected by a Rigaku SmartLab 9kW X-ray Diffractometer. Scanning electron microscopy (SEM) images were obtained on a JEOL JSM IT500 scanning electron microscope. Transmission electron microscopy (TEM) images were performed on Tecnai G2 F30, JEOL 2100F, and JEM-ARM300F2 transmission electron microscopes. X-ray photoelectron spectroscopy (XPS) measurements were conducted on the ESCALAB Xi+ (Thermo Fisher) instrument. C 1s peak (284.8 eV) was used to correct the binding energy of other elements. Diffusion reflection spectroscopy measurements were performed on a Hitachi UH4150 UV-VIS-NIR Spectrophotometer. Raman spectra were measured by using a WITec alpha300 R Raman System with the 633 nm laser source. Synchrotron-based X-ray absorption fine structure (XAFS) of Pb L_3-edge is collected on the BL14B2 beamline at the SPring8 Japan Synchrotron Radiation Facility. Photoluminescence (PL) and photoluminescence excitation (PLE) measurements were carried out on a Hitachi F-4600 spectrophotometer equipped with an R3788 photomultiplier. Luminescence decay curves were recorded using the FLS1000 spectrometer (Edinburgh Instrument Ltd., U.K.) equipped with a μF900 flash lamp and 372.5 nm picosecond pulsed diode laser as pulse light source, and the lifetime data were fitted by the FAST program integrated in the FLS1000.

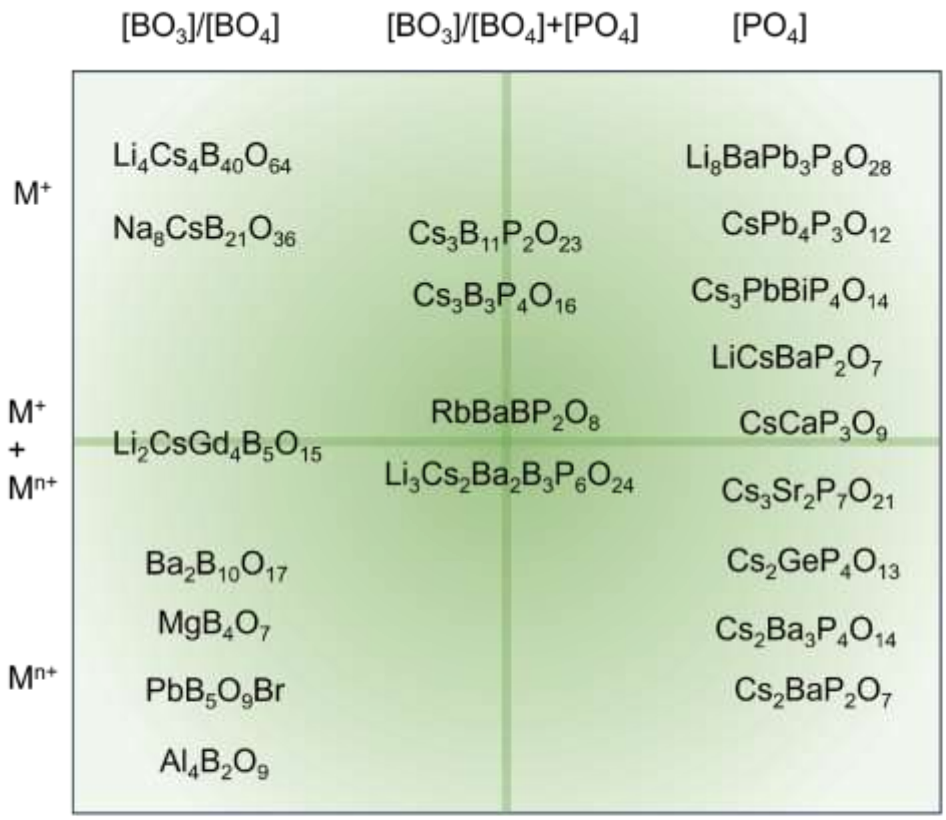

Figure S1. The series of compounds collected by the combination of different large cations (M^+ and M^{n+} represent the monovalent and multivalent cations, respectively) and small-cation units (including $[BO_3]$, $[BO_4]$, and $[PO_4]$).

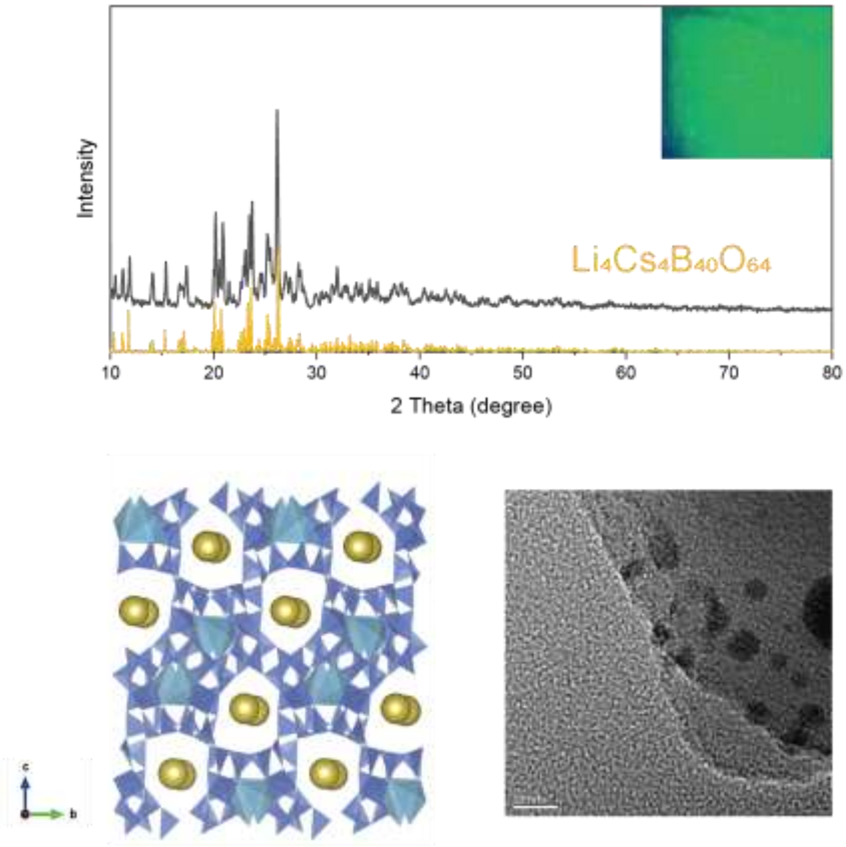

Figure S2. XRD pattern, corresponding crystal structure diagram, and TEM image of $Li_4Cs_4B_{40}O_{64}$-CPB. The inset in the XRD spectrum is the photograph of the material under 365 nm UV light.

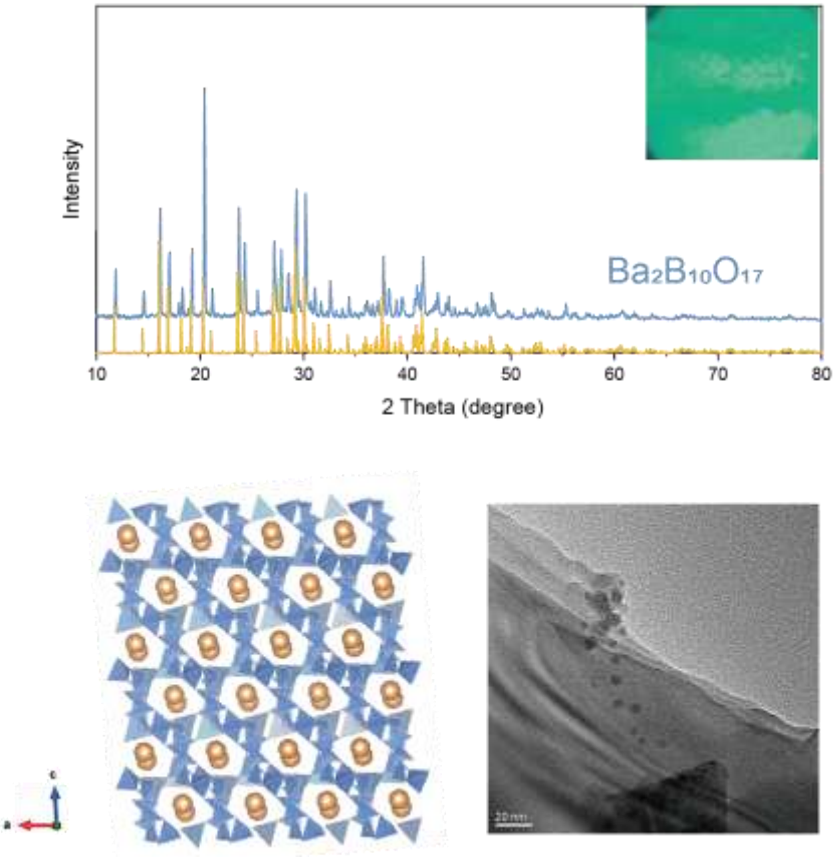

Figure S3. XRD pattern, corresponding crystal structure diagram, and TEM image of $Ba_2B_{10}O_{17}$-CPB. The inset in the XRD spectrum is the photograph of the material under 365 nm UV light.

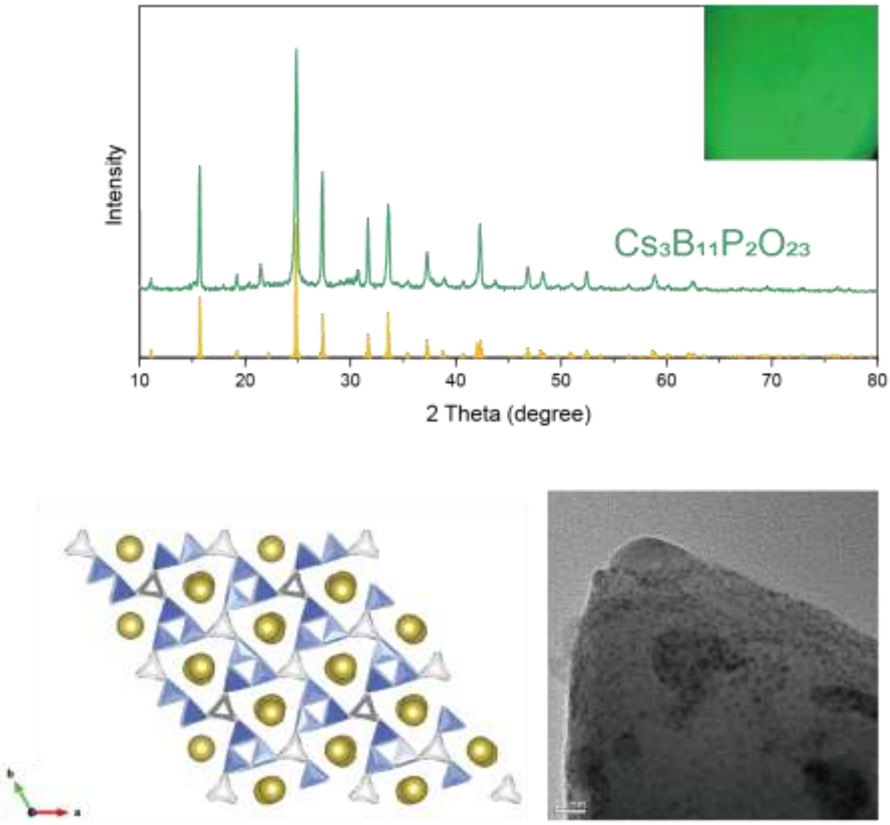

Figure S4. XRD pattern, corresponding crystal structure diagram, and TEM image of $Cs_3B_{11}P_2O_{23}$-CPB. The inset in the XRD spectrum is the photograph of the material under 365 nm UV light.

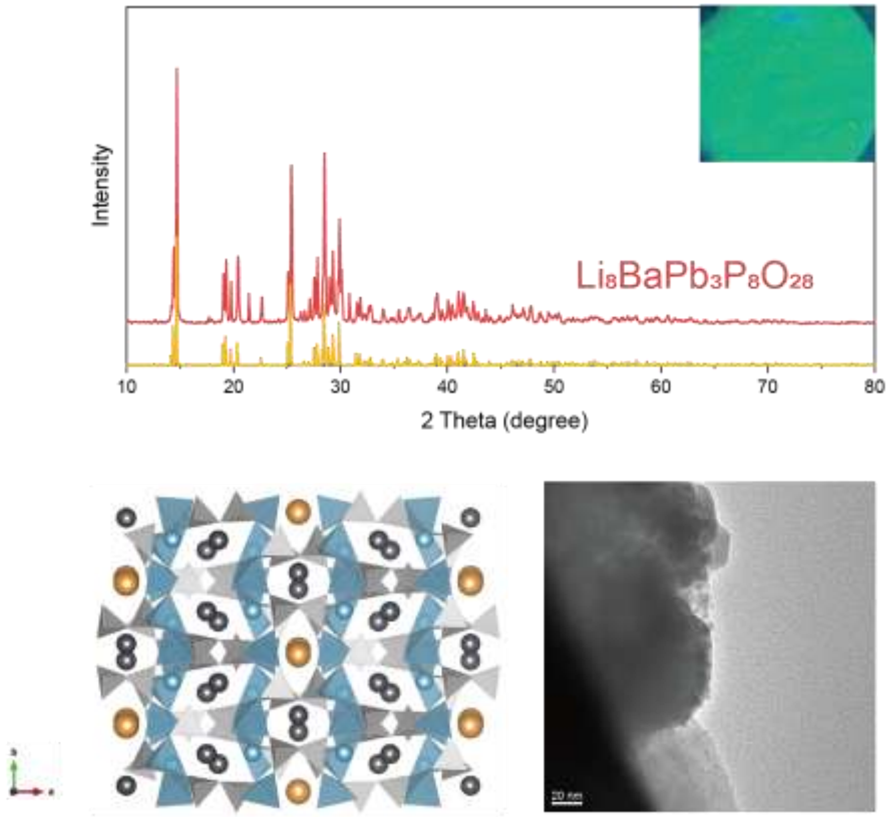

Figure S5. XRD pattern, corresponding crystal structure diagram, and TEM image of $Li_8BaPb_3P_8O_{28}$-CPB. The inset in the XRD spectrum is the photograph of the material under 365 nm UV light.

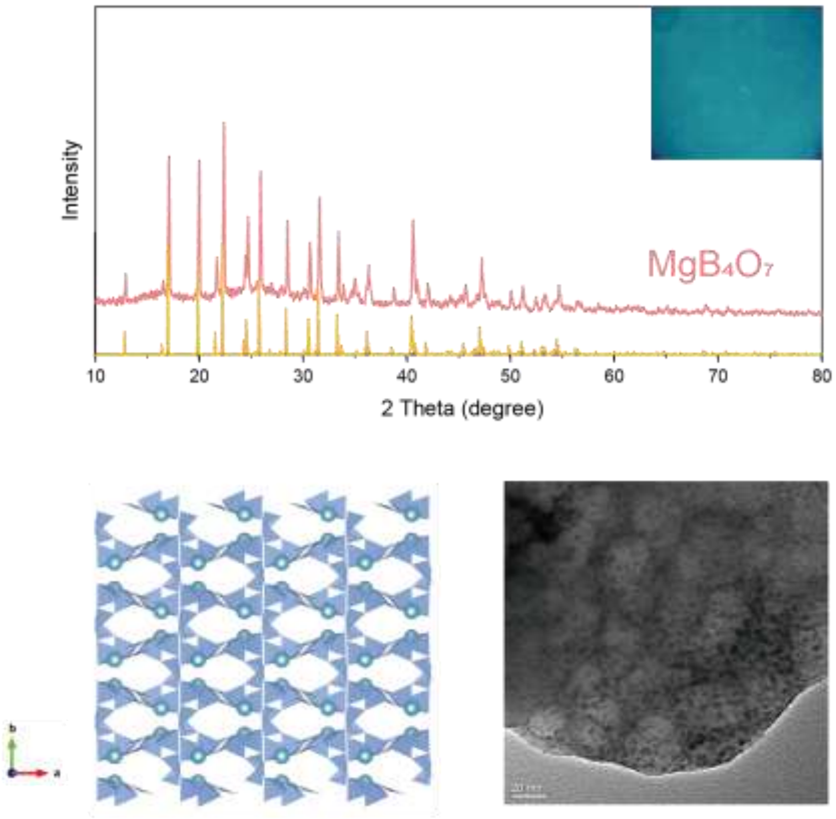

Figure S6. XRD pattern, corresponding crystal structure diagram, and TEM image of MgB_4O_7-CPB. The inset in the XRD spectrum is the photograph of the material under 365 nm UV light.

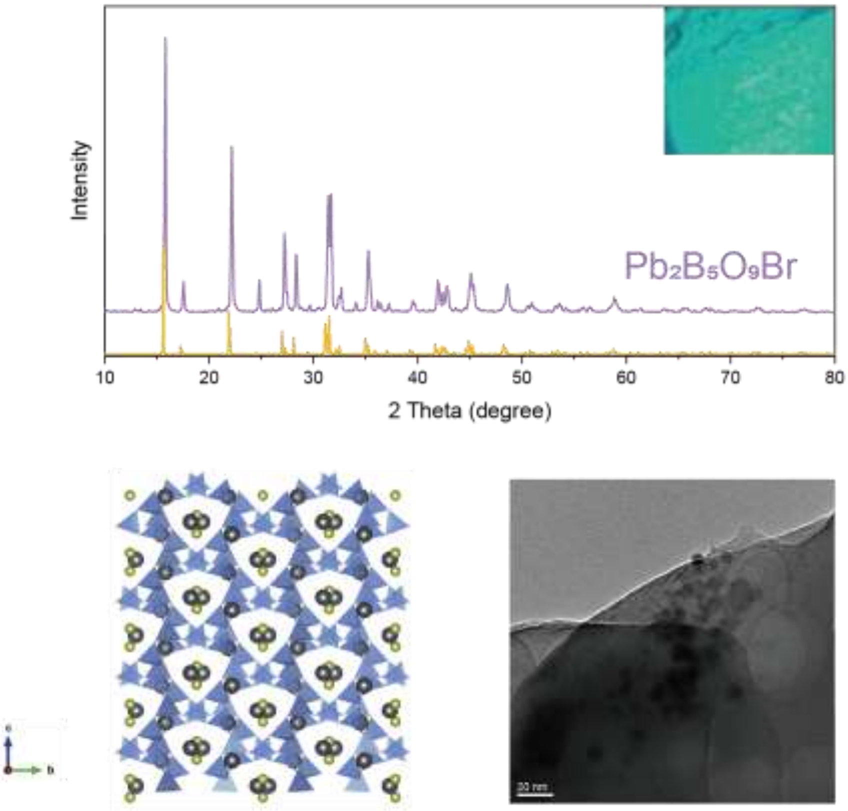

Figure S7. XRD pattern, corresponding crystal structure diagram, and TEM image of PbB_5O_9Br-CPB. The inset in the XRD spectrum is the photograph of the material under 365 nm UV light.

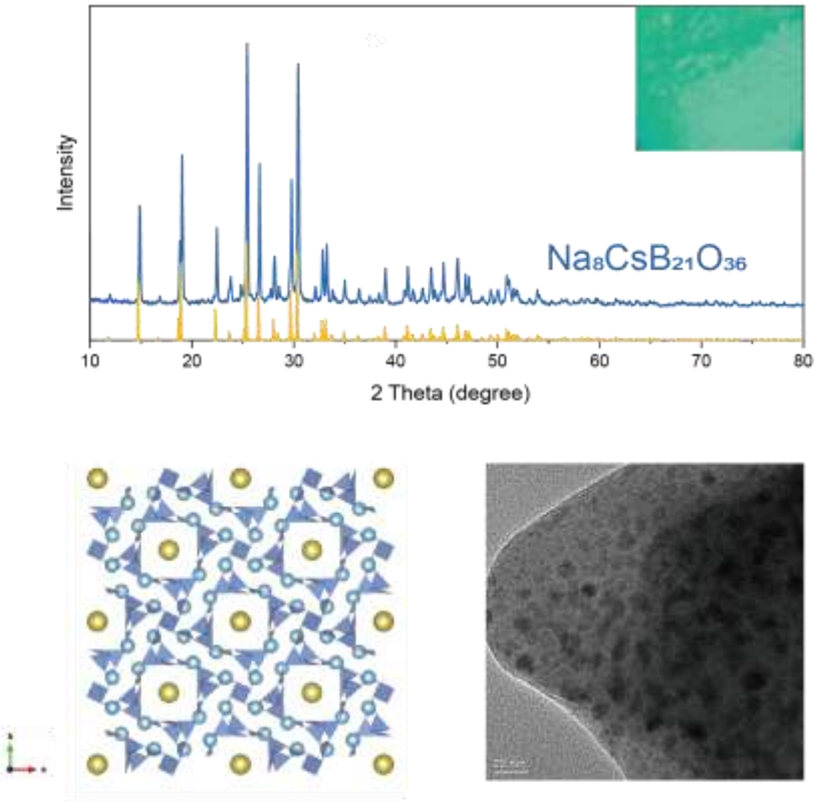

Figure S8. XRD pattern, corresponding crystal structure diagram, and TEM image of $Na_8CsB_{21}O_{36}$-CPB. The inset in the XRD spectrum is the photograph of the material under 365 nm UV light.

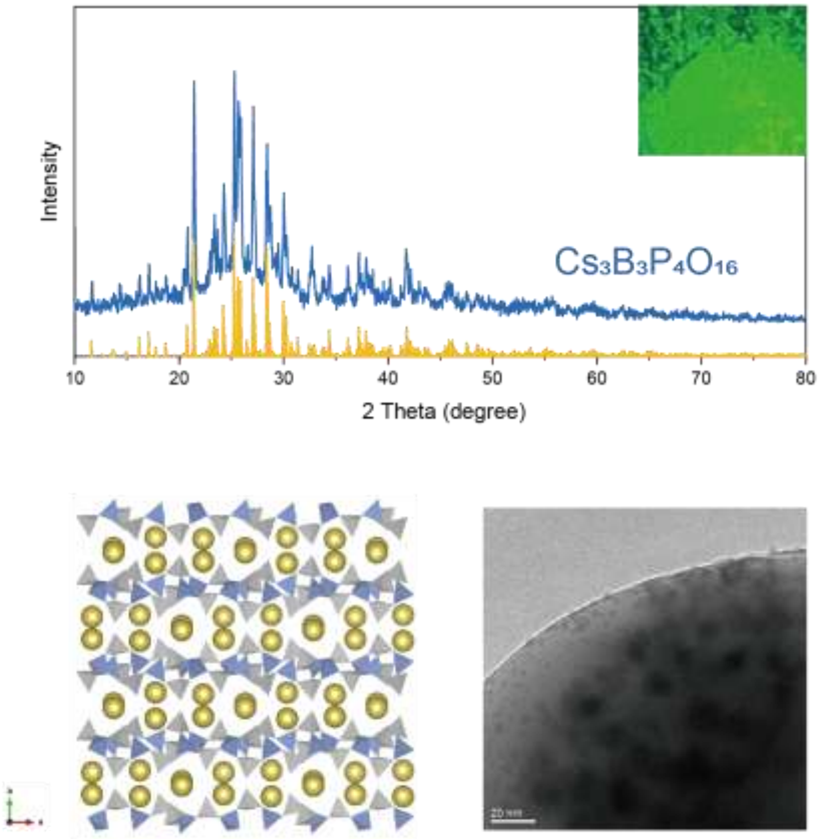

Figure S9. XRD pattern, corresponding crystal structure diagram, and TEM image of $Cs_3B_3P_4O_{16}$-CPB. The inset in the XRD spectrum is the photograph of the material under 365 nm UV light.

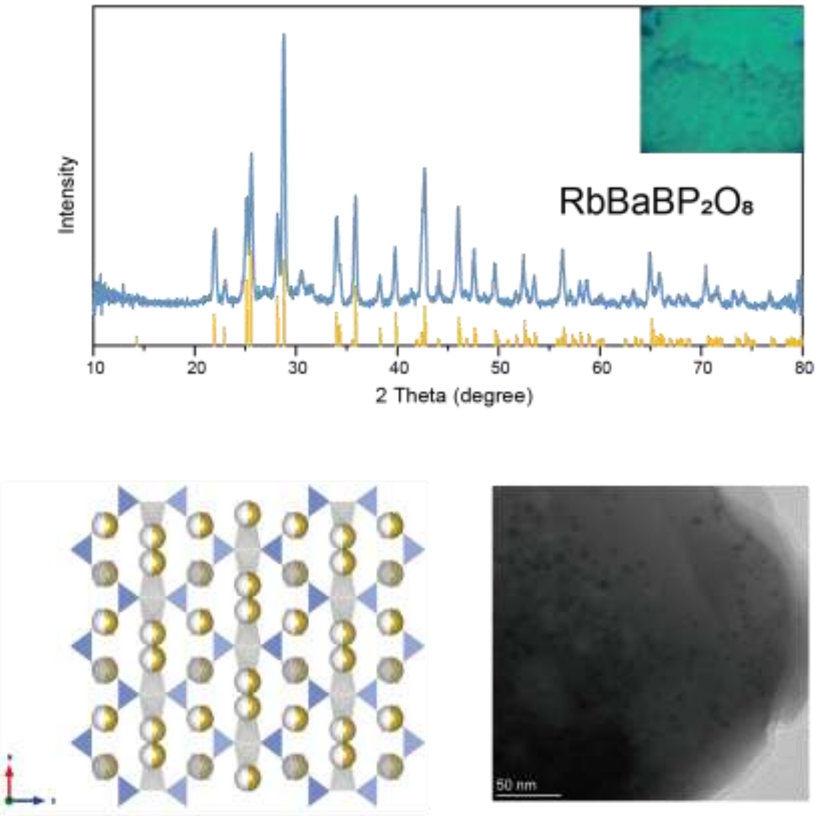

Figure S10. XRD pattern, corresponding crystal structure diagram, and TEM image of $RbBaBP_2O_8$-CPB. The inset in the XRD spectrum is the photograph of the material under 365 nm UV light.

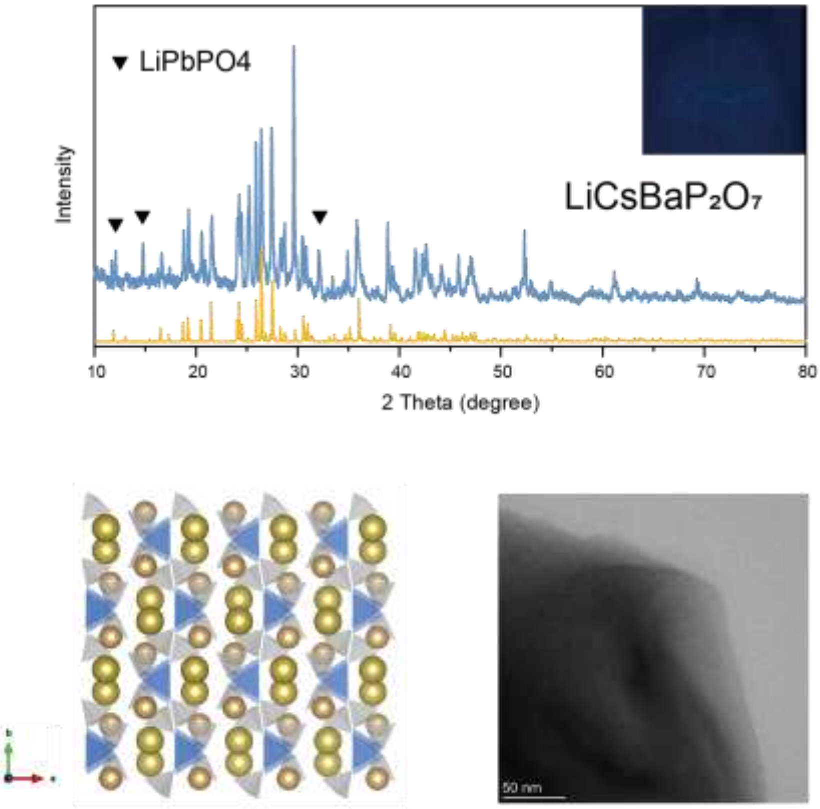

Figure S11. XRD pattern, corresponding crystal structure diagram, and TEM image of $LiCsBaP_2O_7$. The inset in the XRD spectrum is the photograph of the material under 365 nm UV light.

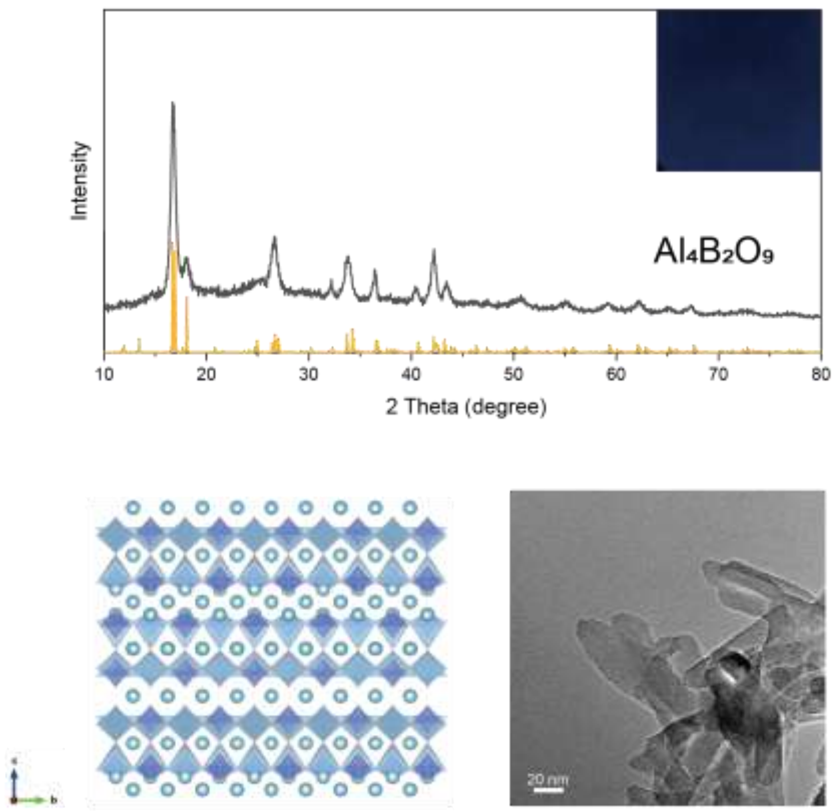

Figure S12. XRD pattern, corresponding crystal structure diagram, and TEM image of $Al_4B_2O_9$. The inset in the XRD spectrum is the photograph of the material under 365 nm UV light.

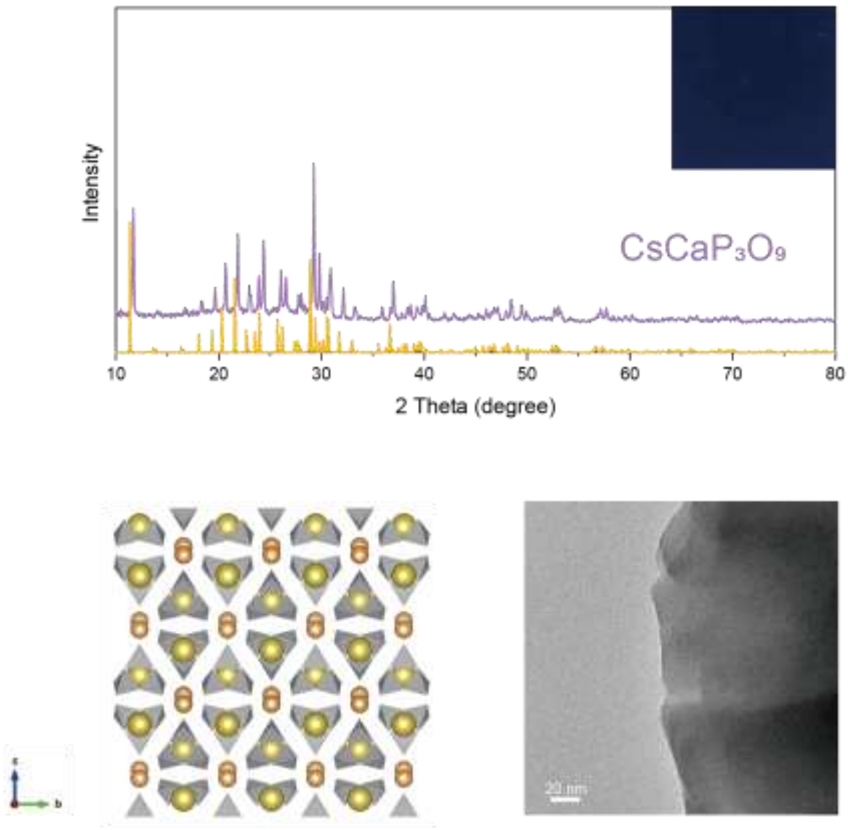

Figure S13. XRD pattern, corresponding crystal structure diagram, and TEM image of $CsCaP_3O_9$. The inset in the XRD spectrum is the photograph of the material under 365 nm UV light.

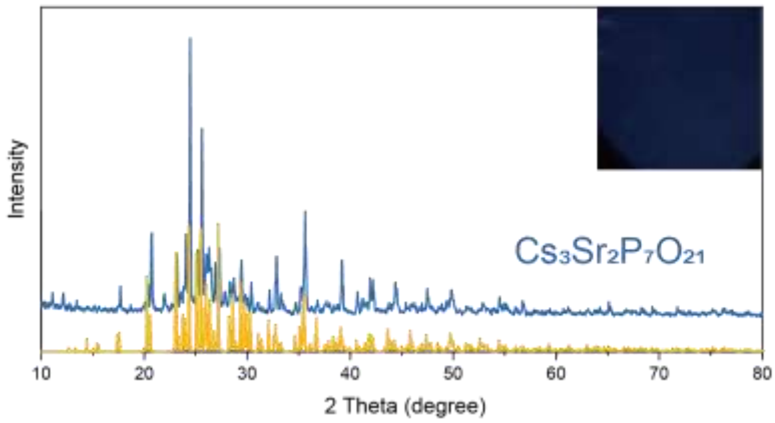

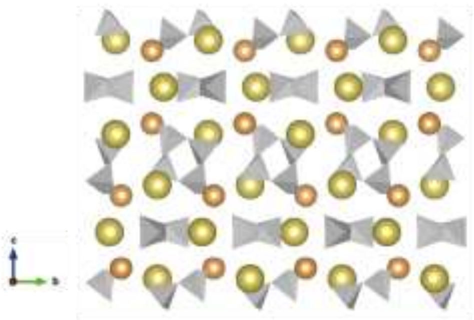

Figure S14. XRD pattern and corresponding crystal structure diagram of $Cs_3Sr_2P_7O_{21}$. The inset in the XRD spectrum is the photograph of the material under 365 nm UV light.

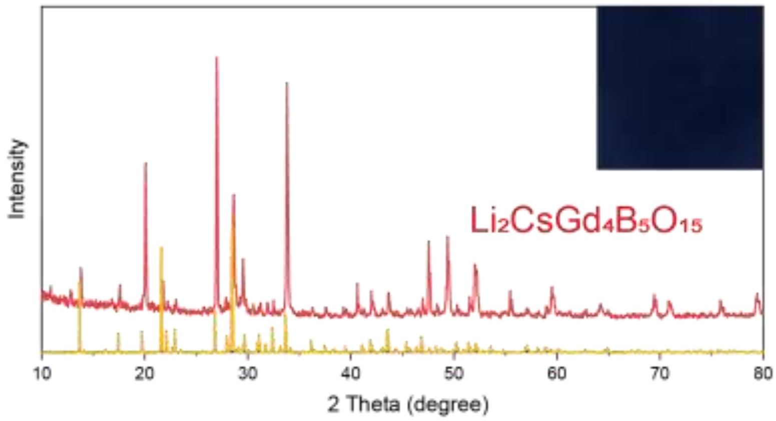

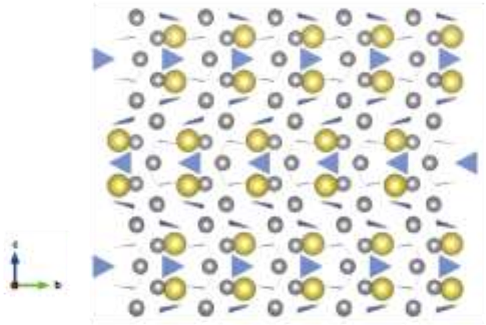

Figure S15. XRD pattern and corresponding crystal structure diagram of $Li_2CsGd_4B_5O_{15}$. The inset in the XRD spectrum is the photograph of the material under 365 nm UV light.

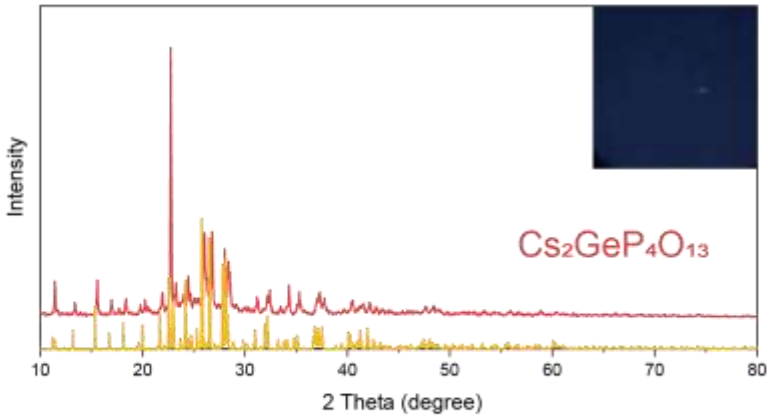

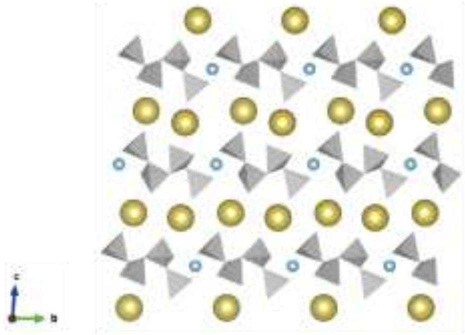

Figure S16. XRD pattern and corresponding crystal structure diagram of $Cs_2GeP_4O_{13}$. The inset in the XRD spectrum is the photograph of the material under 365 nm UV light.

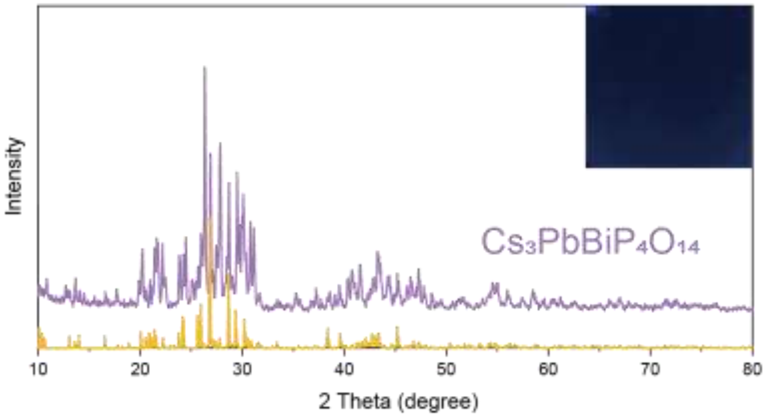

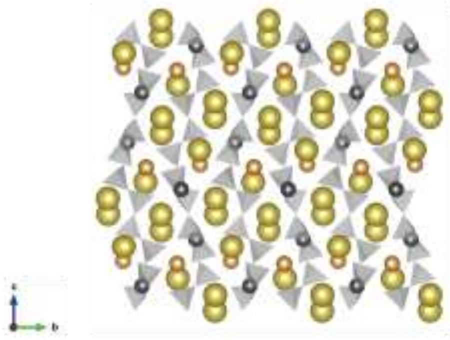

Figure S17. XRD pattern and corresponding crystal structure diagram of $Cs_3PbBiP_4O_{14}$. The inset in the XRD spectrum is the photograph of the material under 365 nm UV light.

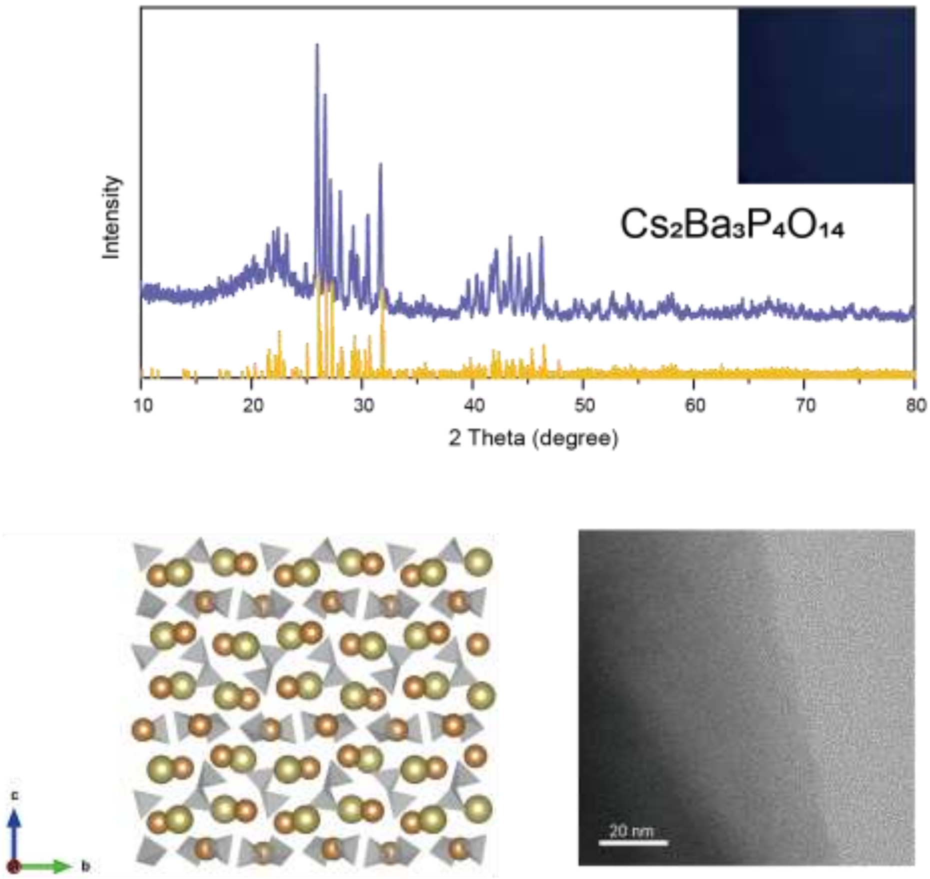

Figure S18. XRD pattern, corresponding crystal structure diagram, and TEM image of $Cs_2Ba_3P_4O_{14}$. The inset in the XRD spectrum is the photograph of the material under 365 nm UV light.

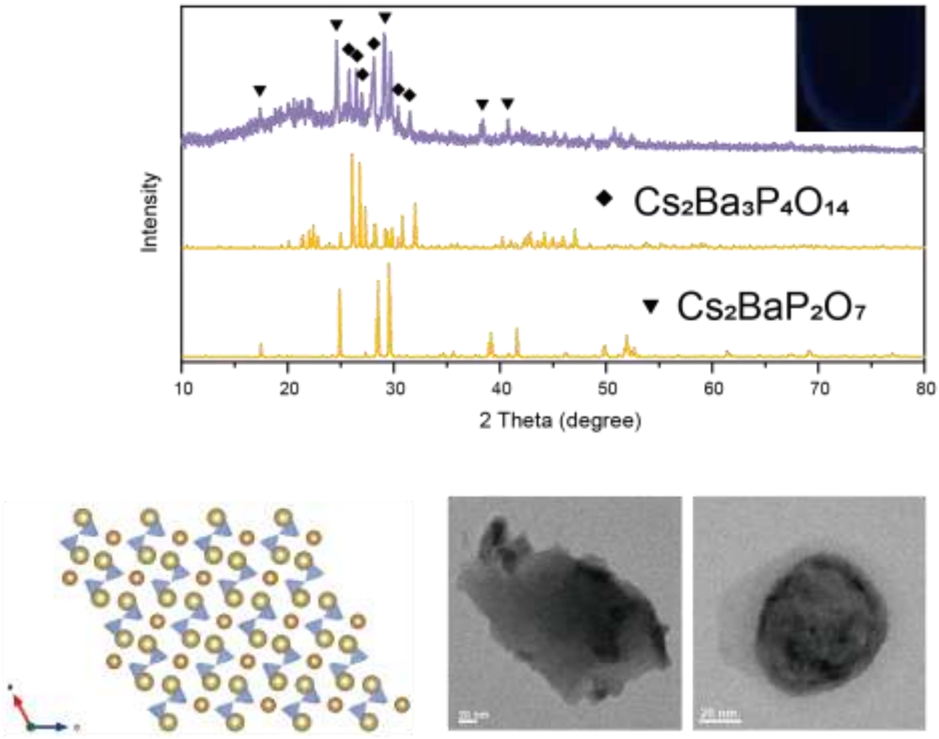

Figure S19. XRD pattern, corresponding crystal structure diagram, and TEM image of $Cs_2BaP_2O_7$. The inset in the XRD spectrum is the photograph of the material under 365 nm UV light.

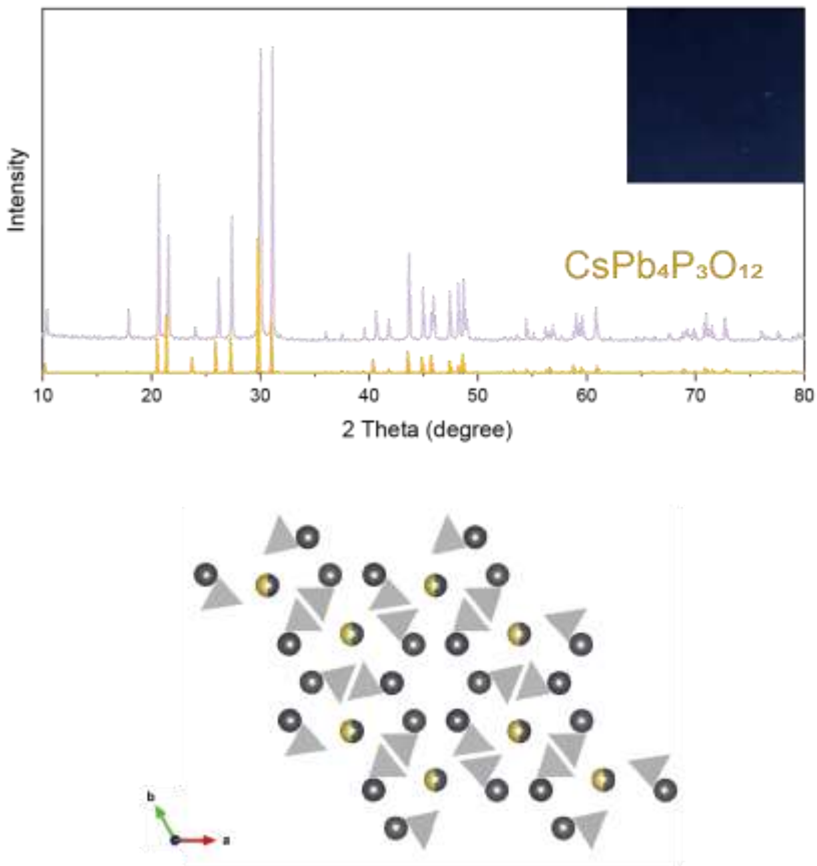

Figure S20. XRD pattern and corresponding crystal structure diagram of $CsPb_4P_3O_{12}$. The inset in the XRD spectrum is the photograph of the material under 365 nm UV light.

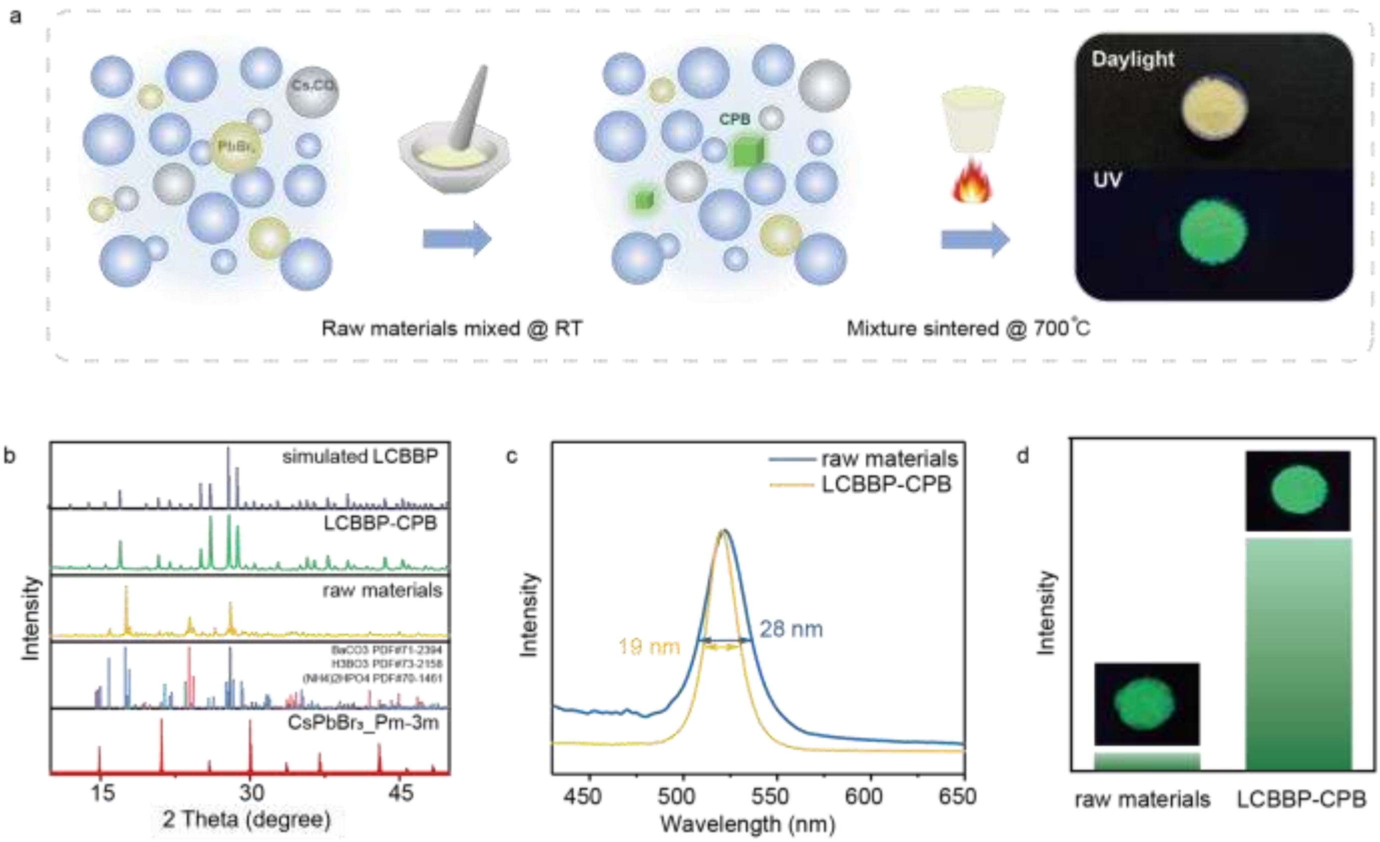

Figure S21. (a) Schematic illustration of the synthesis of host-CPB materials, using LCBBP-CPB as an example. (b) XRD patterns of the materials before and after calcination, along with the standard reference patterns. (c) PL spectra and (d) relative PL intensities measured under 365 nm excitation for the materials before and after calcination.

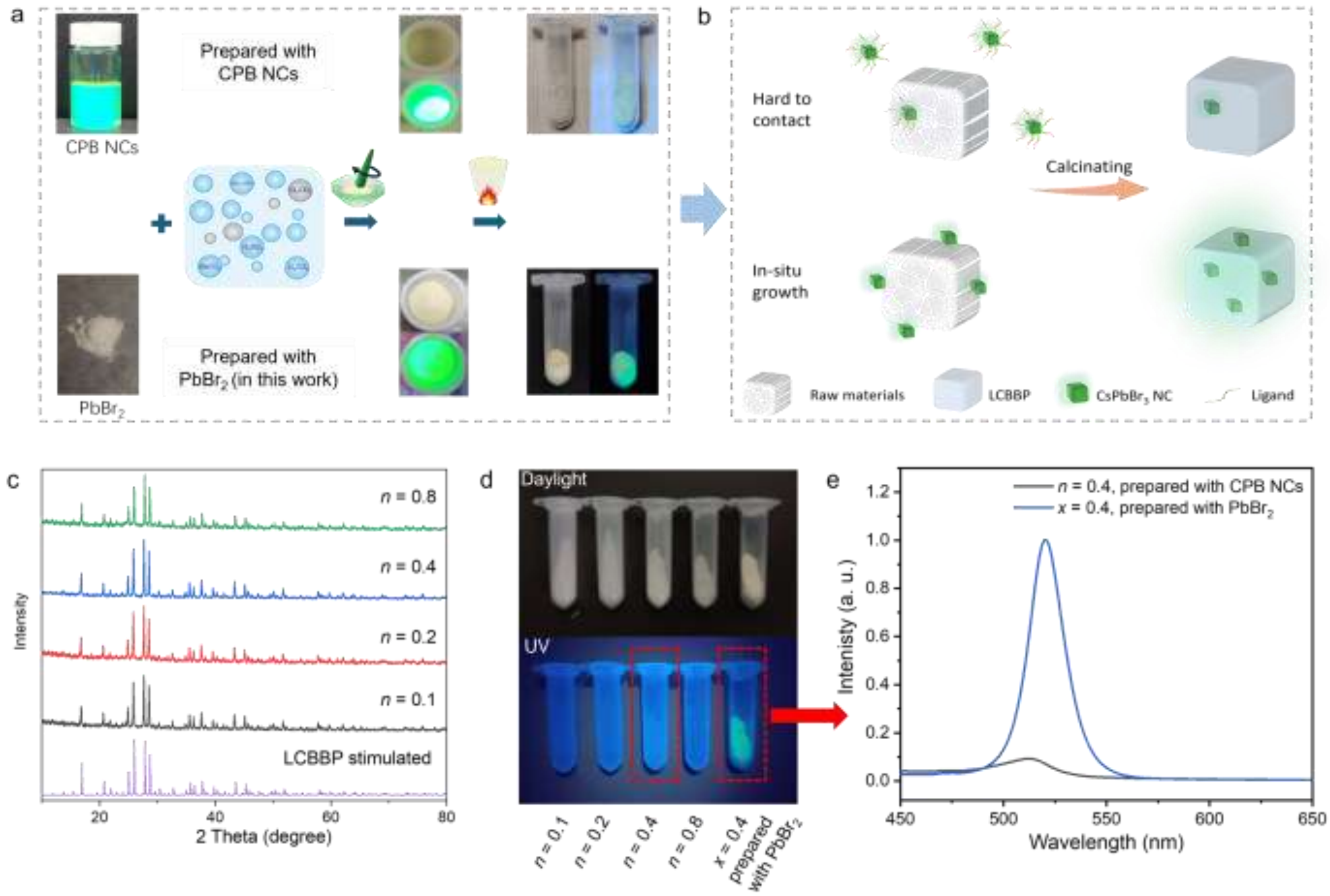

Figure S22. Comparison of incorporating $CsPbBr_3$ nanocrystals (CPB NCs) into the LCBBP matrix via two different precursor routes (a) and the corresponding mechanism scheme (b): (1) adding pre-synthesized colloidal CPB NCs to the raw materials, and (2) the in-situ growth method used in this work (prepared with $PbBr_2$). All samples were calcined at 700 °C for 1 hour. (c) XRD patterns of samples prepared with CPB NCs of varying molar amounts (n). (d) Photographs of samples in (c) along with the sample prepared with $PbBr_2$ (x = 0.4) as a reference, captured under daylight and UV excitation. (e) Comparison of emission spectra between samples prepared with CPB NCs (n = 0.4) and that prepared with $PbBr_2$ (x = 0.4).

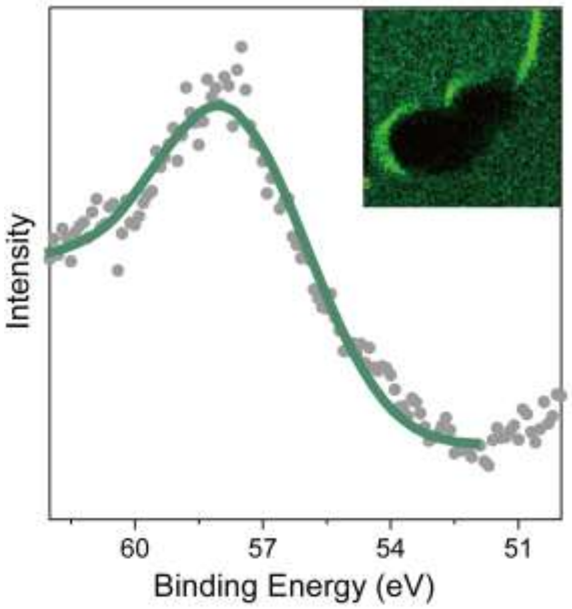

Figure S23. High-resolution XPS profile at Li 1s position for LCBBP-CPB. The inset displays the EDS elemental mapping of Li, revealing no detectable signals.

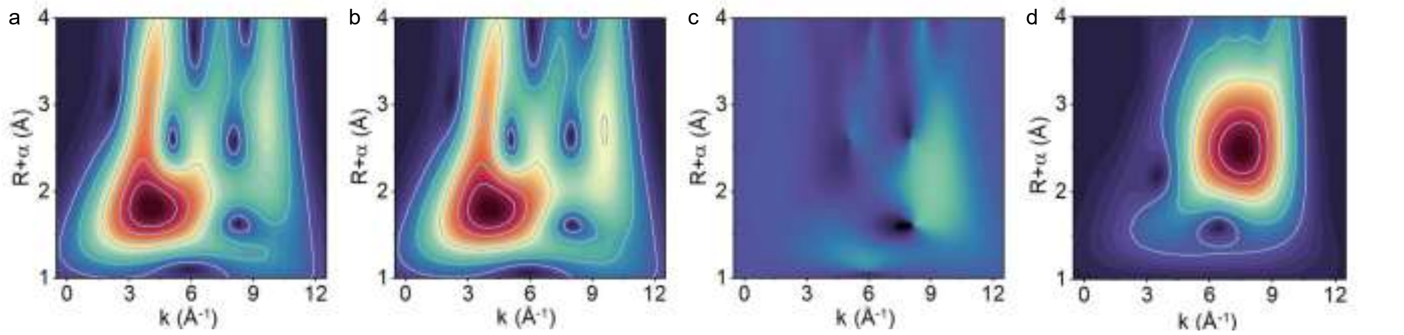

Figure S24. Wavelet transform of the k^3 weighted EXAFS data of LCBBP:Pb (a), LCBBP-CPB (b), the difference of the first two (c), and $CsPbBr_3$ NCs (d), respectively.

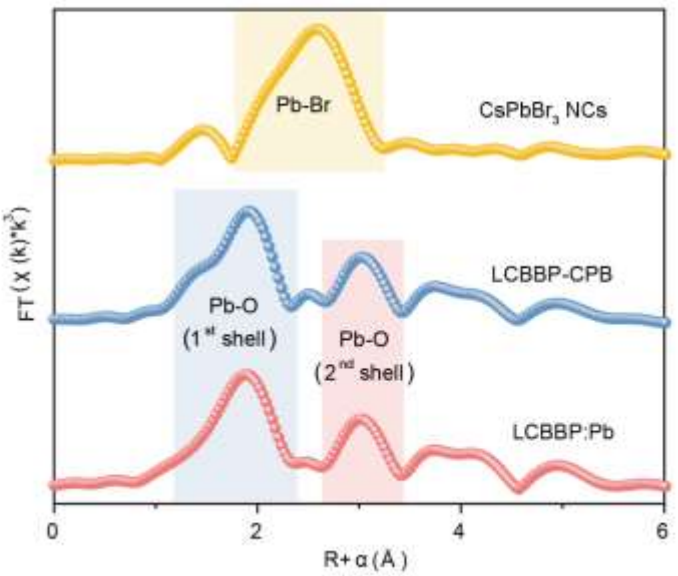

Figure S25. The k^3 weighted Pb L_3-edge EXAFS spectra of $CsPbBr_3$ NCs, LCBBP-CPB, and LCBBP:Pb, respectively.

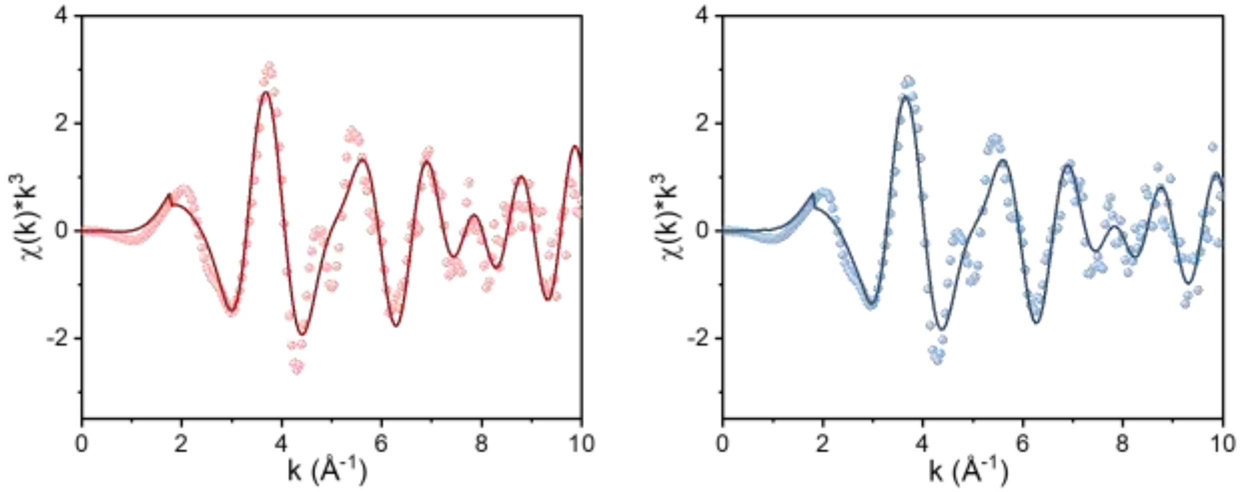

Figure S26. k^3-weighted EXAFS data in k space (balls) at the Pb-L_3 edge for LCBBP:Pb (left) and LCBBP-CPB (right), along with corresponding fittings (solid lines).

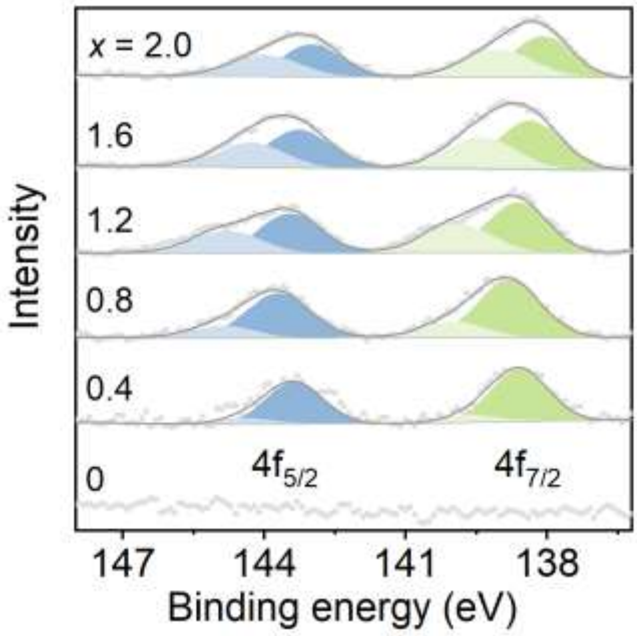

Figure S27. XPS spectra for $Li_3Cs_2Ba_{2-x}Pb_xB_3P_6O_{24}$-CPB with various *x* mol of $PbBr_2$ as raw material.

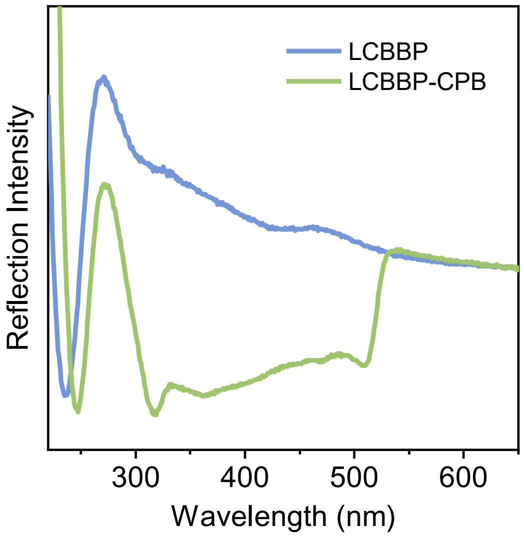

Figure S28. DRS spectra for LCBBP and LCBBP-CPB.

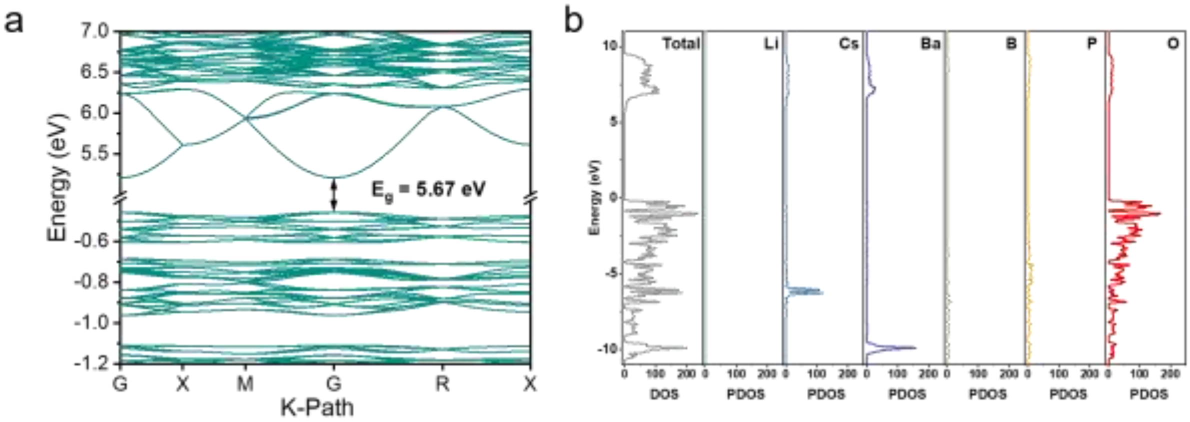

Figure S29. Calculated Band structure (a) and density of states (b) of LCBBP. The calculation was carried out in the framework of DFT combined with the projector-augmented wave method, as implemented in the Vienna Ab Initio Simulation Package (VASP) code. The Perdew–Burke–Ernzerhof function was applied to guarantee the accuracy of the calculations. The cutoff energy of the plane wave was set as 550 eV throughout the simulation, and a 2 × 2 × 2 k-point mesh in the Brillouin zone was employed. The band structure of bare LCBBP was calculated by the HSE hybrid function using primitive cells. The structure was optimized until the force between each atom was smaller than 0.01 eV/Å. The VESTA package was used to plot crystal structures.

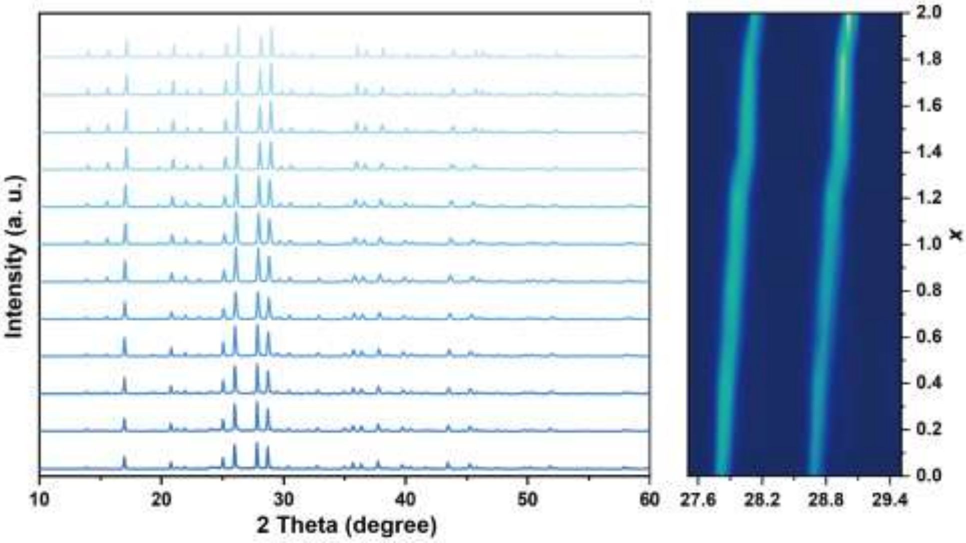

Figure S30. XRD patterns for $Li_3Cs_2Ba_{2-x}Pb_xB_3P_6O_{24}$-CPB with various *x* mol of $PbBr_2$ as raw material. The peak position moves to a higher degree with increasing Pb substitution.

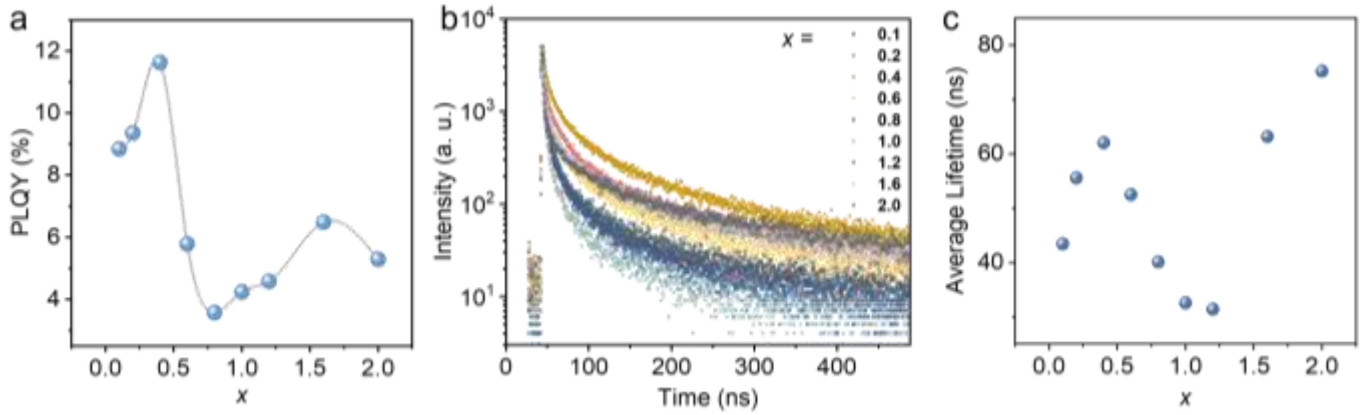

Figure S31. The PLQY (a), fluorescence decay curves (b), and calculated decay lifetimes (c) of $Li_3Cs_2Ba_{2-x}Pb_xB_3P_6O_{24}$-CPB with varying molar amounts (x) of $PbBr_2$ as raw material.

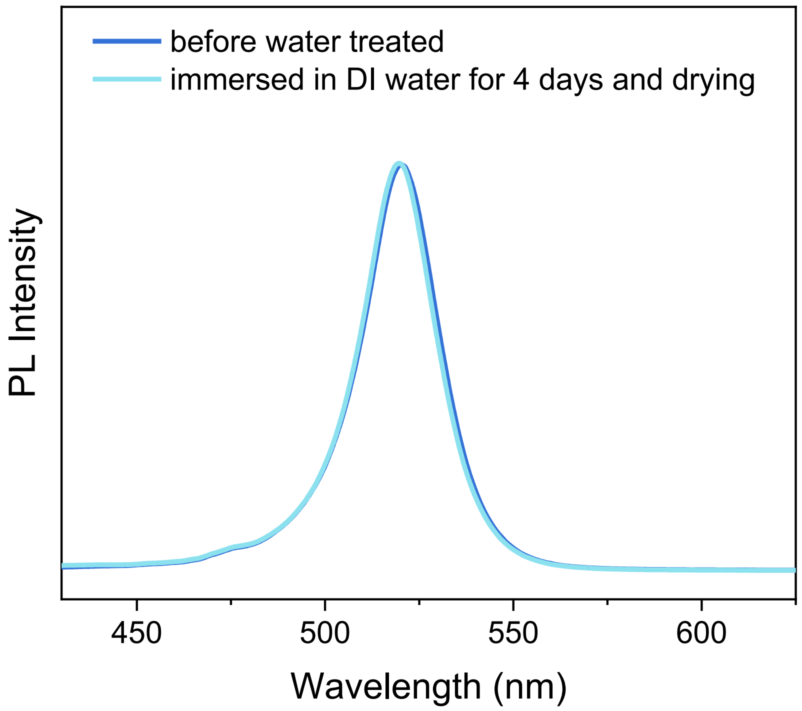

Figure S32. PL spectra for LCBBP-CPB before treatment and after immersing in DI water for 4 days and drying.

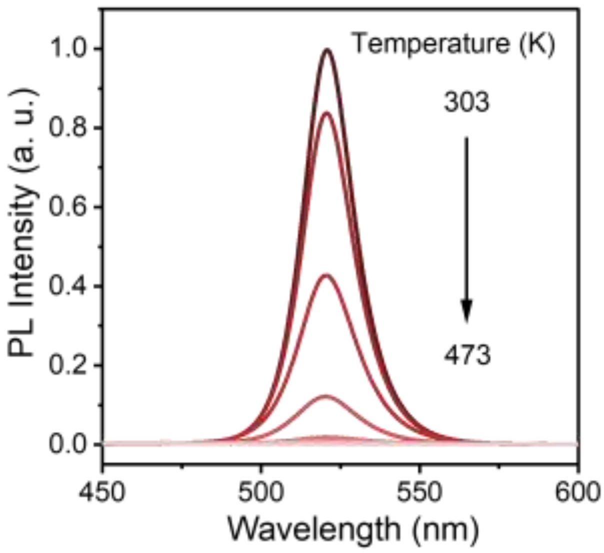

Figure S33. Temperature-dependent PL spectra of the $CsPbBr_3$ NCs.

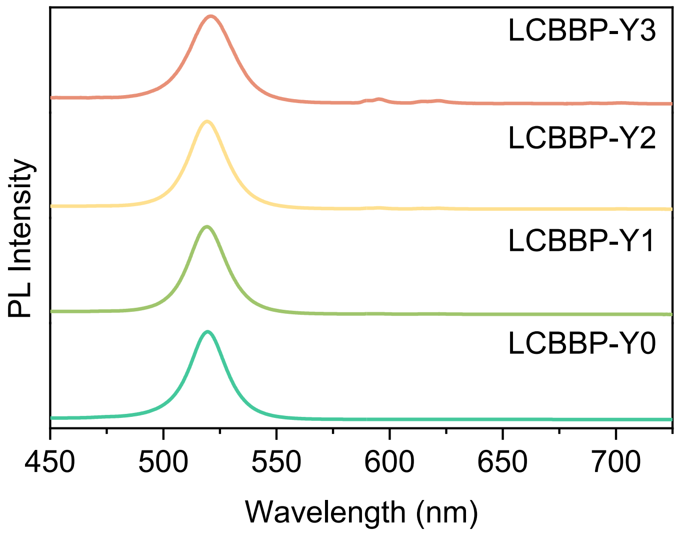

Figure S34. PL spectra for LCBBP-CPB: *y* Eu^{3+} (*y* = 0, 0.1, 0.2, and 0.3 (denoted as LCBBP-Y0, Y1, Y2, and Y3, respectively) excited under 365 nm.

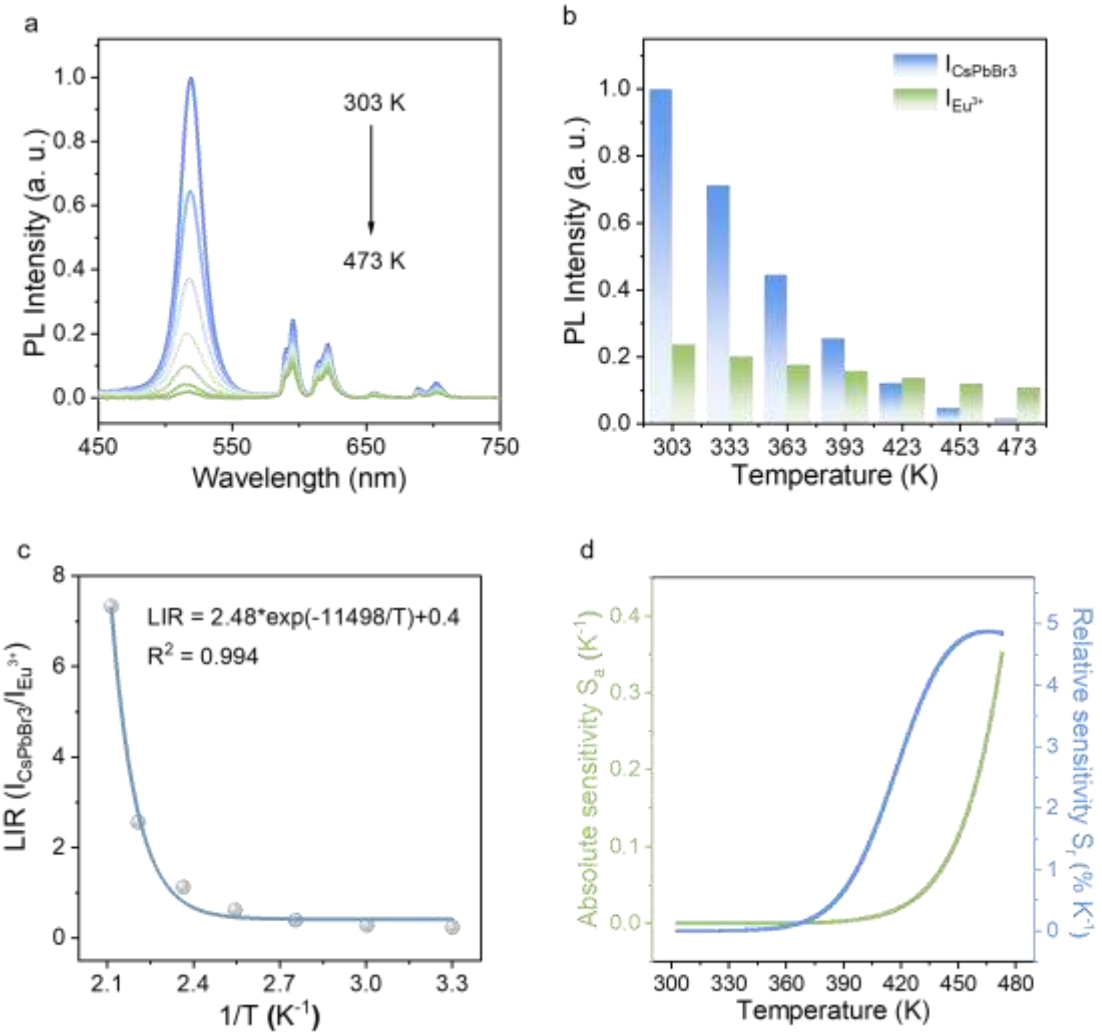

Figure S35. Temperature-dependent luminescence performances of LCBBP-Y1 under 254 nm excitation, corresponding to Figure 5d. (a) Temperature-dependent PL spectra. (b) The integral PL intensity of the green emission from $CsPbBr_3$ NCs and the red emission from Eu^{3+} as a function of heating temperature. (c) The evolution of luminescence intensity ratio (LIR) with temperature. (d) The fitting curve of S_r (blue) and S_a (green) as a function of temperature from 303 to 473 K in LIR mode. The maximum S_r of 4.87% K^{-1} is obtained at 463 K.

Table S1. Chemicals and synthesis conditions for host-CPB materials.*

No.	Chemical formula	Raw materials	
		For host	For $CsPbBr_3$**
**1**	$Li_4Cs_4B_{40}O_{64}$		$PbBr_2$
**2**	$Ba_2B_{10}O_{17}$		CsBr, $PbBr_2$
**3**	$Cs_3B_{11}P_2O_{23}$		$PbBr_2$
**4**	$Li_8BaPb_3P_8O_{28}$		CsBr, $PbBr_2$
**5**	MgB_4O_7		CsBr, $PbBr_2$
**6**	$Li_3Cs_2Ba_2B_3P_6O_{24}$		$PbBr_2$
**7**	PbB_5O_9Br		CsBr, $PbBr_2$
**8**	$Na_8CsB_{21}O_{36}$		$PbBr_2$
**9**	$Cs_3B_3P_4O_{16}$	Li_2CO_3, Cs_2CO_3, $BaCO_3$, H_3BO_3, $(NH_4)_2HPO_4$, $PbCO_3$, Na_2CO, $CaCO_3$, GeO, $SrCO_3$, Gd_2O_3, Bi_2O_3, Al_2O_3, MgO	$PbBr_2$
**10**	$RbBaBP_2O_8$		$PbBr_2$
**11**	$LiCsBaP_2O_7$		$PbBr_2$
**12**	$Al_4B_2O_9$		CsBr, $PbBr_2$
**13**	$CsCaP_3O_9$		$PbBr_2$
**14**	$Cs_3Sr_2P_7O_{21}$		$PbBr_2$
**15**	$Li_2CsGd_4B_5O_{15}$		$PbBr_2$
**16**	$Cs_2GeP_4O_{13}$		$PbBr_2$
**17**	$Cs_3PbBiP_4O_{14}$		$PbBr_2$
**18**	$Cs_2Ba_3P_4O_{14}$		$PbBr_2$
**19**	$Cs_2BaP_2O_7$		$PbBr_2$
**20**	$CsPb_4P_3O_{12}$		$PbBr_2$

* All the calcinated samples were washed with deionized water to prevent possible misjudgment of the results.

** For samples with Cs-contained raw materials, only $PbBr_2$ was added to generate $CsPbBr_3$; for samples without Cs-contained raw materials, CsBr and $PbBr_2$ were added. The relative ratio of raw materials for the host was reduced accordingly to ensure the final mixture was in stoichiometric proportion. For those without Cs or Pb, the amount of additional CsBr and $PbBr_2$ was decreased to avoid impurity formation.

All materials were synthesized using a similar protocol: heating rate of 5 °C/min, holding for 1 hour at 550–900 °C, and naturally cooling down to room temperature.

Table S2. The data for correlation analysis.

Oxides	L/S	MD(LP)	MD(d)	PF	Density (g/cm^3)	H*
$Li_4Cs_4B_{40}O_{64}$	0.09091	0.98880	0.99998	0.54287	2.42	1
$Ba_2B_{10}O_{17}$	0.2	0.87494	0.99975	0.64993	3.52	1
$Cs_3B_{11}P_2O_{23}$	0.23077	0.96063	0.99990	0.61546	3.06	1
$Li_8BaPb_3P_8O_{28}$	0.25	0.91591	0.99953	0.55280	4.2	1
MgB_4O_7	0.25	0.96358	0.99989	0.62216	2.53	1
$Li_3Cs_2Ba_2B_3P_6O_{24}$	0.33333	0.91392	0.99966	0.60457	3.64	1
$Pb_2B_5O_9Br$	0.4	0.97798	0.99976	0.64660	5.34	1
$Na_8CsB_{21}O_{36}$	0.42857	0.89311	0.99963	0.57164	2.5	1
$Cs_3B_3P_4O_{16}$	0.42857	0.95243	0.99996	0.58132	3.42	1
$RbBaBP_2O_8$	0.66667	0.85921	0.89480	0.58957	3.7	1
$LiCsBaP_2O_7$	0.66667	0.93489	0.99662	0.50578	3.62	1
$Al_4B_2O_9$	0.71429	0.94009	0.99940	0.61374	2.94	0
$CsCaP_3O_9$	0.66667	0.91838	0.99995	0.50741	2.86	0
$Cs_3Sr_2P_7O_{21}$	0.71429	0.94154	0.99999	0.56479	3.49	0
$Li_2CsGd_4B_5O_{15}$	0.71429	0.90832	0.99985	0.59767	5.33	0
$Cs_2GeP_4O_{13}$	0.75	0.94187	0.99999	0.59316	3.66	0
$Cs_3PbBiP_4O_{14}$	1.25	0.99482	0.99992	0.56276	4.81	0
$Cs_2Ba_3P_4O_{14}$	1.25	0.94280	0.99997	0.55704	4.21	0
$Cs_2BaP_2O_7$	1.5	0.93607	0.97086	0.55378	4.09	0
$CsPb_4P_3O_{12}$	1.66667	0.89101	0.99998	0.55439	6.42	0

H* “1” represents the successful construction of host-CPB heterostructure, and “0” represents that no heterostructure was obtained and no photoluminescence was observed.

Table S3. Interpretation of the Pearson correlation coefficient. The Pearson correlation coefficient (*r*) quantifies the strength and direction of a linear relationship between two continuous variables, with values ranging from +1 (perfect positive correlation) to -1 (perfect negative correlation). A value near zero indicates the absence of a linear association.

Value of *r*	Interpretation
**+1**	**Perfect positive correlation:** as one variable increases, the other increases in a fixed proportion.
**-1**	**Perfect negative correlation:** as one variable increases, the other decreases in a fixed proportion.
**0**	**No linear correlation:** there is no linear relationship between the two variables.
**0 < *r* < 1**	**Positive correlation:** the closer the value is to 1, the stronger the positive correlation.
**-1 < *r* < 0**	**Negative correlation:** the closer the value is to -1, the stronger the negative correlation.

In our analysis, the strong negative correlation ($r < -0.75$) between the L/S ratio and heterostructure formation (H) underscores the critical role of the large-to-small cation ratio in facilitating the formation of heterostructures.

Table S4. Calculation of L/S and L/All.

**Structures**	**L**	**S**	**All**	**L/S**	**L/All**
$Li_4Cs_4B_{40}O_{64}$	4	44	112	0.09091	0.03571
$Ba_2B_{10}O_{17}$	2	10	29	0.2	0.06897
$Cs_3B_{11}P_2O_{23}$	3	13	39	0.23077	0.07692
$Li_8BaPb_3P_8O_{28}$	4	16	48	0.25	0.08333
MgB_4O_7	1	4	12	0.25	0.08333
$Li_3Cs_2Ba_2B_3P_6O_{24}$	4	12	40	0.33333	0.1
$Pb_2B_5O_9Br$	2	5	17	0.4	0.11765
$Na_8CsB_{21}O_{36}$	9	21	66	0.42857	0.13636
$Cs_3B_3P_4O_{16}$	3	7	26	0.42857	0.11538
$Al_4B_2O_9$	5	7	30	0.71429	0.16667
$CsCaP_3O_9$	2	3	14	0.66667	0.14286
$Cs_3Sr_2P_7O_{21}$	5	7	33	0.71429	0.15152
$Li_2CsGd_4B_5O_{15}$	5	7	27	0.71429	0.18519
$Cs_2GeP_4O_{13}$	3	4	20	0.75	0.15
$Cs_3PbBiP_4O_{14}$	5	4	23	1.25	0.21739
$Cs_2Ba_3P_4O_{14}$	5	4	23	1.25	0.21739
$RbBaBP_2O_8$	2	3	13	0.66667	0.15385
$LiCsBaP_2O_7$	2	3	12	0.66667	0.16667
$Cs_2BaP_2O_7$	3	2	12	1.5	0.25
$CsPb_4P_3O_{12}$	5	3	20	1.66667	0.25

All: all the ions (cations + anions) in the unit cell.

Table S5. Calculation of MD(LP).

**Structure**	***a***	***b***	***c***	**MD(LP)**
$CsPbBr_3$	5.8733	5.8733	5.8733	/
$Li_4Cs_4B_{40}O_{64}$	11.0442	11.0981	23.7594	0.9888
$Ba_2B_{10}O_{17}$	6.7128	9.8698	9.9998	0.87494
$Cs_3B_{11}P_2O_{23}$	11.3016	11.3016	13.955	0.96063
$Li_8BaPb_3P_8O_{28}$	25.65	9.9763	9.9245	0.91591
MgB_4O_7	13.73	7.97	8.62	0.96358
$Li_3Cs_2Ba_2B_3P_6O_{24}$	12.853	12.853	12.853	0.91392
$Pb_2B_5O_9Br$	11.4935	11.4717	6.5297	0.97798
$Na_8CsB_{21}O_{36}$	15.039	15.039	6.5762	0.89311
$Cs_3B_3P_4O_{16}$	13.0515	15.3256	7.9289	0.95243
$Al_4B_2O_9$	14.8056	5.5413	15.0531	0.94009
$CsCaP_3O_9$	9.8287	7.5642	12.7905	0.91838
$Cs_3Sr_2P_7O_{21}$	6.9636	13.995	22.1957	0.94154
$Li_2CsGd_4B_5O_{15}$	10.644	6.4661	20.093	0.90832
$Cs_2GeP_4O_{13}$	4.9749	7.8498	15.899	0.94187
$Cs_3PbBiP_4O_{14}$	9.4243	9.7261	17.5291	0.99482
$Cs_2Ba_3P_4O_{14}$	9.017	9.606	18.689	0.9428
$CsPb_4P_3O_{12}$	9.9734	9.9734	7.4898	0.89101
$RbBaBP_2O_8$	7.281	7.281	14.33	0.85921
$LiCsBaP_2O_7$	7.18071	10.98439	10.88137	0.93061
$Cs_2BaP_2O_7$	10.92874	6.2744	15.45587	0.93607

Table S6. The Interplanar spacing (d_{i2}) for different host structures.

**Structure**	***i*=1**	***i*=2**	***i*=3**	***i*=4**	***i*=5**	***i*=6**	***i*=7**	***i*=8**	***i*=9**	***i*=10**	***i*=11**	***i*=12**	***i*=13**
$Li_4Cs_4B_{40}O_{64}$	5.7962	4.1385	3.3916	2.9327	2.6251	2.3984	2.0768	1.9578	1.8576	1.7705	1.6958	1.6292	1.5695
$Ba_2B_{10}O_{17}$	5.6688	4.1436	3.3516	2.9428	2.6193	2.3943	2.0718	1.9521	1.8578	1.7701	1.6915	1.624	1.5693
$Cs_3B_{11}P_2O_{23}$	5.6815	4.0065	3.2862	2.8408	2.6646	2.4101	2.1358	1.9535	1.8463	1.7879	1.7046	1.6313	1.5699
$Li_8BaPb_3P_8O_{28}$	5.9794	4.0246	3.4097	2.9897	2.6357	2.3941	2.0741	1.9566	1.8551	1.7725	1.6966	1.63	1.569
MgB_4O_7	5.3834	4.1122	3.4325	2.926	2.6007	2.3812	2.0769	1.9591	1.8597	1.7769	1.6953	1.6292	1.5688
$Li_3Cs_2Ba_2B_3P_6O_{24}$	5.748	4.0645	3.4351	2.9487	2.6236	2.3867	2.085	1.9601	1.8552	1.7655	1.7024	1.6323	1.5702
$Pb_2B_5O_9Br$	5.7468	4.0597	3.4477	2.8734	2.5691	2.3907	2.0836	1.9684	1.8548	1.7844	1.6961	1.6324	1.5701
$Na_8CsB_{21}O_{36}$	6.0253	3.9868	3.3628	2.9494	2.6585	2.4107	2.0842	1.9404	1.848	1.7715	1.6929	1.632	1.5707
$Cs_3B_3P_4O_{16}$	5.9298	4.1619	3.3706	2.9577	2.6342	2.3953	2.0744	1.9597	1.8581	1.7722	1.694	1.6276	1.5696
$Al_4B_2O_9$	5.3197	4.1226	3.3761	2.9533	2.6178	2.3874	2.0785	1.959	1.8596	1.7719	1.6987	1.6257	1.5708
$CsCaP_3O_9$	5.4279	4.121	3.4026	2.9266	2.5978	2.399	2.0713	1.9579	1.8571	1.7721	1.6945	1.6277	1.5747
$Cs_3Sr_2P_7O_{21}$	5.9021	4.3209	3.3856	2.951	2.6372	2.3948	2.077	1.9644	1.8543	1.7709	1.6928	1.631	1.5695
$Li_2CsGd_4B_5O_{15}$	6.1339	4.1119	3.3959	2.9703	2.6257	2.3994	2.076	1.9575	1.8569	1.7674	1.6944	1.6319	1.5683
$Cs_2GeP_4O_{13}$	5.7692	4.1411	3.3954	2.9508	2.6273	2.3972	2.0764	1.9591	1.8592	1.7719	1.6953	1.629	1.57
$Cs_3PbBiP_4O_{14}$	6.3139	4.1503	3.3841	2.9284	2.6388	2.4049	2.0752	1.9582	1.8616	1.7699	1.6969	1.6295	1.5696
$Cs_2Ba_3P_4O_{14}$	6.2018	4.1484	3.4139	2.9441	2.6134	2.4015	2.0784	1.9543	1.8609	1.7707	1.6954	1.6295	1.5698
$CsPb_4P_3O_{12}$	5.6586	4.1509	3.4359	2.9926	2.6874	2.3984	2.0754	1.9815	1.8706	1.7642	1.6836	1.6323	1.5697
$RbBaBP_2O_8$	6.4912	4.181	3.2456	3.1752	2.6668	2.3929	2.0905	1.9707	1.86	1.7642	1.6689	1.6281	1.5648
$LiCsBaP_2O_7$	5.8535	4.2352	3.4064	2.9389	2.6243	2.3897	2.0861	1.9586	1.8598	1.7717	1.6975	1.6282	1.5697
$Cs_2BaP_2O_7$	5.7021	4.1015	3.376	2.9117	2.6312	2.3987	2.0781	1.9573	1.8539	1.7677	1.6952	1.6286	1.5693

Table S7. Calculation of MD(d).

Structure	MD(d)
$Li_4Cs_4B_{40}O_{64}$	0.99998
$Ba_2B_{10}O_{17}$	0.99975
$Cs_3B_{11}P_2O_{23}$	0.99990
$Li_8BaPb_3P_8O_{28}$	0.99953
MgB_4O_7	0.99989
$Li_3Cs_2Ba_2B_3P_6O_{24}$	0.99966
$Pb_2B_5O_9Br$	0.99976
$Na_8CsB_{21}O_{36}$	0.99963
$Cs_3B_3P_4O_{16}$	0.99996
$Al_4B_2O_9$	0.99940
$CsCaP_3O_9$	0.99995
$Cs_3Sr_2P_7O_{21}$	0.99999
$Li_2CsGd_4B_5O_{15}$	0.99985
$Cs_2GeP_4O_{13}$	0.99999
$Cs_3PbBiP_4O_{14}$	0.99992
$Cs_2Ba_3P_4O_{14}$	0.99997
$CsPb_4P_3O_{12}$	0.99998
$RbBaBP_2O_8$	0.89480
$LiCsBaP_2O_7$	0.99662
$Cs_2BaP_2O_7$	0.97086

Table S8. Calculation of PF.

**Structure**	$\boldsymbol{V}_{\mathbf{cell}}$	$\sum \boldsymbol{V}_{\mathbf{ion}}$	**PF**
$Li_4Cs_4B_{40}O_{64}$	2764.2500	1500.631	0.54287
$Ba_2B_{10}O_{17}$	617.0600	401.0444	0.64993
$Cs_3B_{11}P_2O_{23}$	1543.6100	950.028	0.61546
$Li_8BaPb_3P_8O_{28}$	2390.8200	1321.648	0.55280
MgB_4O_7	943.2700	586.8604	0.62216
$Li_3Cs_2Ba_2B_3P_6O_{24}$	2123.3100	1283.7	0.60457
$Pb_2B_5O_9Br$	860.94	556.6802	0.64660
$Na_8CsB_{21}O_{36}$	1487.3400	850.2302	0.57164
$Cs_3B_3P_4O_{16}$	1576.4200	916.4117	0.58132
$Al_4B_2O_9$	1234.8300	757.866	0.61374
$CsCaP_3O_9$	950.9300	482.514	0.50741
$Cs_3Sr_2P_7O_{21}$	2141.4400	1209.455	0.56479
$Li_2CsGd_4B_5O_{15}$	1334.2100	797.4133	0.59767
$Cs_2GeP_4O_{13}$	608.1800	360.7493	0.59316
$Cs_3PbBiP_4O_{14}$	1606.7400	904.2115	0.56276
$Cs_2Ba_3P_4O_{14}$	1618.7900	901.7386	0.55704
$CsPb_4P_3O_{12}$	645.1800	357.6803	0.55439
$RbBaBP_2O_8$	759.6758	447.8787	0.58957
$LiCsBaP_2O_7$	826.8121	418.186	0.50578
$Cs_2BaP_2O_7$	937.0995	518.9476	0.55378

Table S9. Rietveld refinement results for LCBBP, basic information

**Composition**	$Li_3Cs_2Ba_2B_3(PO_4)_6$
**Crystal system**	Cubic
**Space group**	$P2_13$ (*No.* 198)
**Lattice parameters**	$a = b = c$ = 12.8429(0) Å , $\alpha = \beta = \gamma$ = 90˚
Z	4
**Cell volume**	2118.29(0) $Å^3$
R_p	2.00%
R_{wp}	2.96%
χ^2	2.57

Table S10. Rietveld refinement results for LCBBP, atomic parameters

Atomic parameters

Atom	Wyck.	x/a	y/b	z/c	U($Å^2$)
**Ba1**	4a	0.3417(2)	0.1582(8)	0.8417(2)	0.0184
**Ba2**	4a	0.1681(5)	0.6681(5)	0.8318(5)	0.0116
**Cs1**	4a	0.4016(9)	0.5983(1)	1.0983(1)	0.0277
**Cs2**	4a	0.6055(9)	0.3944(1)	0.8944(1)	0.0284
**Li1**	12b	0.8168(9)	0.4568(7)	0.6332(8)	0.0984
**B1**	12b	1.0949(1)	0.3789(4)	0.8959(0)	0.0294
**P1**	12b	0.8915(0)	0.3990(7)	0.8641(2)	0.0135
**P2**	12b	0.0673(1)	0.1652(0)	0.8107(5)	0.0091
**O1**	12b	0.8358(0)	0.5065(3)	0.8763(9)	0.0169
**O2**	12b	0.1459(5)	0.0820(4)	0.8572(7)	0.0218
**O3**	12b	0.1141(0)	0.2744(7)	0.8175(6)	0.0414
**O4**	12b	-0.0403(1)	0.1570(9)	0.8569(3)	0.0233
**O5**	12b	0.8684(4)	0.3579(9)	0.7593(4)	0.0539
**O6**	12b	0.8582(9)	0.3342(3)	0.9499(9)	0.0444
**O7**	12b	1.0102(1)	0.4270(9)	0.8747(1)	0.0236
**O8**	12b	1.1859(7)	0.4475(5)	0.8506(3)	0.0157

Table S11. Curvefit parameters [a] for Pb L_3-edge EXAFS for LCBBP:Pb

**Path**	***N*** [b]	**ΔE_0 / eV**	***d*** [c] **/ Å**	***R* / Å**	**σ^2 / Å^2**
**Pb-O6**	3	-3 (1) [d]	2.558	2.343(7)	0.005(1)
**Pb-O2**	3	-3 (1) [d]	2.684	2.514(7)	0.003(1)
**Pb-Li**	3	3	2.885	2.945	0.001
**Pb-O3**	3	12	3.505	3.497	0.001

[a] S_0^2 was fixed as 0.95. Data ranges: $2.3 \leqslant k \leqslant 7.5$ Å^{-1}, $1.0 \leqslant R \leqslant 3.4$ Å. The number of variable parameters is 5, out of a total of 7.7 independent data points. R factor for this fit is 1.2 %. [b] The coordination numbers were constrained as *N*(Pb-O6), *N*(Pb-O2), *N*(Pb-Li), and *N*(Pb-O3) = 3, based on the crystal structure. [c] The distances for Pb-O (6, 2, 3) and Pb-Li are from the FEFF file of LCBBP20 (refined). [d] ΔE_0 was constrained as ΔE_0 (Pb-O6) = ΔE_0 (Pb-O2) to decrease the correlation (or reduce the number of variables).

Table S12. Curvefit parameters [a] for Pb L_3-edge EXAFS for LCBBP-CPB

**Path**	***N*** [b]	**ΔE_0 / eV**	***d*** [c] **/ Å**	***R* / Å**	**σ^2 / Å^2**
**Pb-O6**	3	-4 (1) [d]	2.558	2.34(2)	0.007(2)
**Pb-O2**	3	-4 (1) [d]	2.684	2.50(3)	0.004
**Pb-Li**	4	3	2.885	2.99(2)	0.002
**Pb-O3**	3	12 (1)	3.505	3.51	0.004
**Pb-Br**	1	5	2.974	3.00	0.006

[a] S_0^2 was fixed as 0.95 for Pb-O (6, 2, 3), 0.84 for Pb-Li, and 0.35 for Pb-Br, respectively. Data ranges: $2.3 \leqslant k \leqslant 10$ Å^{-1}, $1.0 \leqslant R \leqslant 3.4$ Å. The number of variable parameters is 6, out of a total of 10.7 independent data points. R factor for this fit is 1.4 %. [b] The coordination numbers were constrained as *N*(Pb-O6), *N*(Pb-O2), *N*(Pb-O3) = 3, *N*(Pb-Li) = 4, and *N*(Pb-Br) = 1 based on the crystal structure. (This is because the fitting was mainly used for verifying the occupied site for Pb in LCBBP-CPB is 6- or 9-coordinated; no more concern was taken on the specific coordination number of Pb). [c] The distances for Pb-O (6, 2, 3), and Pb-Li are from the FEFF file of $Li_3Cs_2Ba_{2-x}Pb_xB_3P_6O_{24}$-CPB ($x$ = 2.0, refined), and Pb-Br from the FEFF file of $CsPbBr_3$ (*Pm-3m*, No.221), respectively. [d] ΔE_0 was constrained as ΔE_0 (Pb-O6) = ΔE_0 (Pb-O2) to decrease the correlation (or reduce the number of variables). Note that the Pb-Br path got a small S_0^2 value because of its low contribution compared to other paths.

Table S13. Refined coordination details for LCBBP and CPB/LCBBP

LCBBP				LCBBP-CPB			
	Atom	Number	Distance (Å)		Atom	Number	Distance (Å)
Ba1	O6	3x	2.6852	Ba1	O2	3x	2.6459
	O2	3x	2.7056		O6	3x	2.7021
	O3	3x	3.2968		Li1	3x	3.1445
	Li1	3x	3.3081		O3	3x	3.4116
	P2	3x	3.5477		P2	3x	3.5365
	P1	3x	3.9017		P1	3x	3.8859
Ba2	O5	3x	2.7453	Ba2	O5	3x	2.627
	O8	3x	2.8526		O8	3x	2.7528
	O4	3x	2.9315		O4	3x	3.0062
	P2	3x	3.5355		P2	3x	3.5479
	Li1	3x	3.7399		Li1	3x	3.6102
	O7	3x	3.7419		O7	3x	3.7349
	B1	3x	3.9188		B1	3x	3.7825
	P1	3x	3.9644		P1	3x	3.9712

Calculation for MD(LP)

For an oxide with lattice parameters a_i, b_i, c_i and CPB with lattice parameter a, the match degree for each lattice vector is calculated as:

$$MD(a) = 1 - \frac{|a_i - n * a|}{a_i} \quad \text{(Equation 1)}$$

$$MD(b) = 1 - \frac{|b_i - n * a|}{b_i} \quad \text{(Equation 2)}$$

$$MD(c) = 1 - \frac{|c_i - n * a|}{c_i} \quad \text{(Equation 3)}$$

where n is a multiplier that can be an integer (1, 2, 3...) or $\sqrt{2}$ (to account for diagonal matches, as suggested in several epitaxy studies (*Adv. Mater.* **2001**, *13*, 227; *Angew. Chem. Int. Ed.* ***2022***, *61*, e202209703; *ACS Nano*. **2021**, *15*, 3415). MD(LP) is then taken as the maximum value among MD(a), MD(b), and MD(c):

$$MD(LP) = \max\{MD(a), MD(b), MD(c)\} \quad \text{(Equation 4)}$$

Calculation for MD(D)

For each of the 13 key CPB planes with spacing d_{i1}, the matching degree with a substrate plane of spacing d_{i2} is:

$$MD(d_i) = 1 - \frac{|d_{i1} - d_{i2}|}{d_{i1}} \quad \text{(Equation 5)}$$

MD(d) for the material pair is the maximum value of $MD(d_i)$ found across all compared planes:

$$MD(d) = \max_{1 \leq i \leq 13} MD(d_i) \quad \text{(Equation 6)}$$

Calculation for PF

PF is calculated as the sum of the spherical volumes of all ions in the unit cell divided by the volume of the unit cell itself:

$$PF = \frac{\sum V_{ion}}{V_{cell}} = \frac{\sum \frac{4}{3}\pi r_{ion}^3}{V_{cell}} \quad \text{(Equation 7)}$$

where r_{ion} is the ionic radius of each ion.
